# Biodegradable reduce expenditure bioreactor for augmented sonodynamic therapy via regulating tumor hypoxia and inducing pro-death autophagy

**DOI:** 10.1186/s12951-021-01166-y

**Published:** 2021-12-13

**Authors:** Weijuan Zou, Junnian Hao, Jianrong Wu, Xiaojun Cai, Bing Hu, Zhigang Wang, Yuanyi Zheng

**Affiliations:** 1 Department of Ultrasound in Medicine, Shanghai Institute of Ultrasound in Medicine, Shanghai Jiao Tong University Affiliated Sixth People’s Hospital, Shanghai, 200233 People’s Republic of China; 2 Chongqing Key Laboratory of Ultrasound Molecular Imaging, Ultrasound Department of the Second Affiliated Hospital of Chongqing Medical University, Chongqing, 400010 People’s Republic of China; 3 State Key Laboratory of Oncogenes and Related Genes, School of Medicine, Shanghai Jiao Tong University, Shanghai, 200233 People’s Republic of China

**Keywords:** Sonodynamic therapy, Hypoxia regulation, Pro-death autophagy, Hollow mesoporous organosilica, Biodegradable

## Abstract

**Backgrounds:**

Sonodynamic therapy (SDT) as an emerging reactive oxygen species (ROS)-mediated antitumor strategy is challenged by the rapid depletion of oxygen, as well as the hypoxic tumor microenvironment. Instead of the presently available coping strategies that amplify the endogenous O_2_ level, we have proposed a biodegradable O_2_ economizer to reduce expenditure for augmenting SDT efficacy in the present study.

**Results:**

We successfully fabricated the O_2_ economizer (HMME@HMONs-3BP-PEG, HHBP) via conjugation of respiration inhibitor 3-bromopyruvate (3BP) with hollow mesoporous organosilica nanoparticles (HMONs), followed by the loading of organic sonosensitizers (hematoporphyrin monomethyl ether; HMME) and further surface modification of poly(ethylene glycol) (PEG). The engineered HHBP features controllable pH/GSH/US-sensitive drug release. The exposed 3BP could effectively inhibit cell respiration for restraining the oxygen consumption, which could alleviate the tumor hypoxia conditions. More interestingly, it could exorbitantly elevate the autophagy level, which in turn induced excessive activation of autophagy for promoting the therapeutic efficacy. As a result, when accompanied with suppressing O_2_-consumption and triggering pro-death autophagy strategy, the HHBP could achieve the remarkable antitumor activity, which was systematically validated both in vivo and in vitro assays.

**Conclusions:**

This work not only provides a reduce expenditure means for enduring SDT, but also represents an inquisitive strategy for tumor treatments by inducing pro-death autophagy.

**Graphical Abstract:**

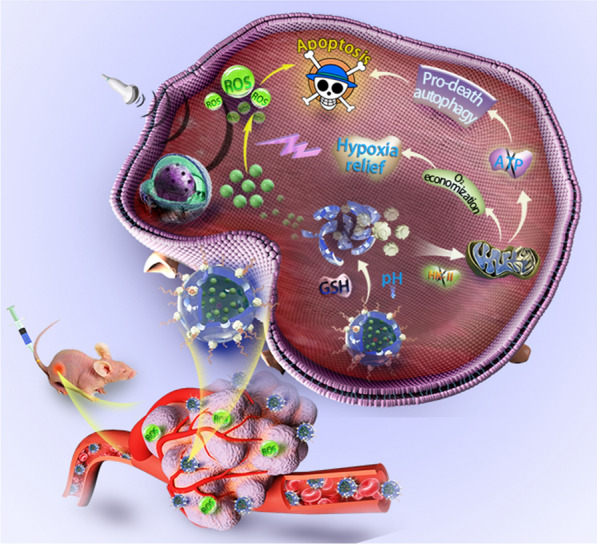

**Supplementary Information:**

The online version contains supplementary material available at 10.1186/s12951-021-01166-y.

## Introduction

As an emerging cancer-treatment modality, sonodynamic therapy (SDT) employs sonosensitizers that are activated by ultrasound (US) in the presence of oxygen, which then enables the generation of highly toxic reactive oxygen species (ROS) in cancer cells, primarily singlet oxygen (^1^O_2_) [1–3]. Due to the deep tissue penetration of the US, SDT offers multiple advantages, such as noninvasiveness and high tissue-penetrating depth relative to that with photodynamic therapy, thereby promising better potential for the treatment of deep-seated tumors [4–7]. Although preclinical researches on SDT have shown promising results in various cancer cells, some undesirable limitations severely restricting the clinical translation of SDT. For instance, the efficacy of SDT mainly depends on oxygen content inside the solid tumors, while several solid tumors often feature hypoxia triggered by the limited oxygen diffusion and the rapid proliferation of cancer cells [8–11]. However, this situation is further challenged by the fact that hypoxia gets further intensified during the SDT process, triggering SDT resistance. Thus, overcoming tumor hypoxia is the priority among priorities in SDT process. In principle, the hypoxic tumor microenvironment (TME) can be reshaped by improving the exogenous O_2_ level or suppressing O_2_ consumption [12–14]. Promoted by the significant advances in nanomedicine, several expectant strategies aimed at modulating the level of oxygen in a solid tumor, such as delivering O_2_ to the hypoxic regions via oxygen-carrying nanomaterials and the in situ generations of O_2_ by nanocatalysts/nanozymes-mediated hydrogen peroxide (H_2_O_2_) decomposition, have been exploited [11, 15–20]. Nevertheless, the feasibility of these strategies is severely hindered by the leakage of O_2_ from the carriers and the insufficient endogenous H_2_O_2_ [3, 21–23]. Compared to the widespread tactics of improving the oxygen content, reducing the oxygen consumption seems like a promising alternative to relieve tumor hypoxia. It has been proved so far that inhibiting mitochondrial respiration by some oxygen-regulator (e.g., NO, [24] atovaquone [25] and metformin [26]) can effectively oxygenate tumors for improving the therapeutic efficacies. Unfortunately, the nanoplatform design that can modulate hypoxia by reducing O_2_ consumption is still necessary, albeit largely unexploited, especially on oxygen-dependent therapeutic modalities.

In addition to induce cell apoptosis, the potential benefit of the production of ^1^O_2_ during the SDT process can induce autophagy [27–29]. Autophagy is a cellular degradation process that refers to the degradation of phagocytes cytoplasmic proteins or organelles through the formation of autophagy lysosomes [30, 31], which commonly considered as a self-protection process aimed at achieving the renewal of some organelles and the metabolism and energy of the cell itself [32]. However, autophagy is a “double-edged sword”. When it exceeds a certain threshold, exorbitant and abnormal autophagy can promote cell death under specific circumstances [33, 34]. It can thus be further hypothesized that the activation of pro-death autophagy can be a potential strategy to improve the efficacy of cancer treatment. Considering that autophagy induced by ROS is pro-survival, the construction of nanosystems with both the sonosensitizer activity and the capability of regulating autophagy from self-protection to pro-death is expected to achieve augmented SDT.

**Fig. 1:**
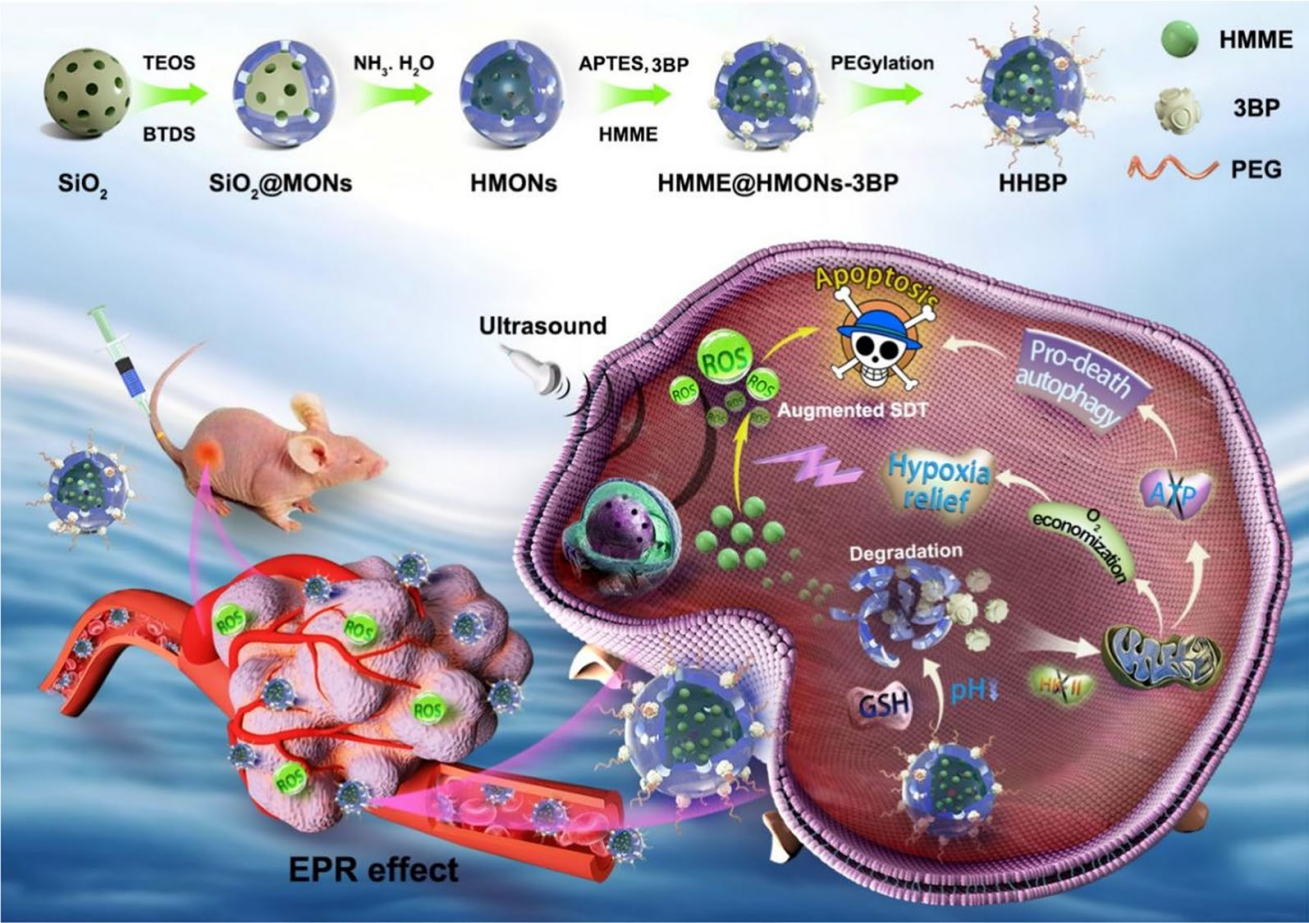
Schematic illustrations of the construction of bioreactor with reduced expenditure based on HHBP nanosonosensitizer and the therapeutic mechanism of the HHBP to enhance the SDT efficacy through the regulation of tumor hypoxia and the induction of pro-death autophagy

Considering the above discussed assumptions, we have proposed a bioreactor with reduced expenditure for augmenting SDT efficacy through the regulation of tumor hypoxia and induction of pro-death autophagy. This bioreactor was constructed by decorating respiration inhibitor 3-bromopyruvate (3BP) onto the surface of disulfide bonds-hybridized hollow mesoporous organosilica nanoparticles (HMONs) via an amide linker. Organic sonosensitizers (hematoporphyrin monomethyl ether, HMME) were further loaded into the mesopores and the hollow interior of HMONs, followed by modification with poly (ethylene glycol) (PEG) (HMME@HMONs-3BP-PEG, designated as HHBP) to enhance their biocompatibility (Fig. 1). The presently engineered HHBP have featured intrinsic TME-responsive biodegradability that function as a nanosonosensitizer to produce ^1^O_2_ under the US irradiation. The delivered 3BP significantly reduces the intracellular oxygen consumption rate by perturbing the respiration process, thus relieving tumor hypoxia for further augmenting the production of ^1^O_2_. Specifically, 3BP as a hexokinase type II (HK-II) inhibitor, can block the first stage of respiration to inhibit both anaerobic and aerobic respiration. Correspondingly, glycolysis was inhibited, which caused the reduction of intracellular production of lactate and adenosine triphosphate (ATP). Therefore, 3BP is expected to shut down the nutrient supply for inhibiting cellular respiration for the rapid depletion of ATP and the simultaneous induction of autophagy [35, 36]. The combination of ROS production and ATP supply restriction results in the changeover of autophagy from self-protection to pro-death, which in turn promotes cell apoptosis. As a result, enhanced SDT therapeutic outcome could be acquired, which was systematically investigated both in vitro at the cellular level and in vivo on 4T1 tumor-bearing mice. These findings demonstrate the feasibility of the present augmented SDT strategy based on the 3BP and HMME co-delivered systems. Our work provides an expenditure reduction strategy for facilitating ROS production, while simultaneously offering a rationale for the combination of respiration inhibition and pro-death autophagy therapy as a therapeutic strategy against cancer.

## Methods

### Synthesis of hollow mesoporous organosilica nanoparticles (HMONs)

The HMONs were prepared as described in our previous work [37, 38]. Briefly, cetyltrimethylammonium chloride (CTAC, 10 g) was dissolved in deionized water (100 mL), to which triethanolamine (8 g) solution was added. The resultant mixture was heated and stirred in an oil bath to 80 °C and tetraethyl orthosilicate (TEOS, 0.5 mL) was added dropwise for 1 h. Then, a mixture of TEOS (1 mL) and bis(3-triethoxysilylpropyl) disulfide (BTDS, 0.6 mL) was immediately added to continue the reaction for 4 h to form the core/shell structure. The products were washed thrice with a mixture of ethanol (50 mL) and hydrochloric acid (4 mL) in a reflux unit at 80 ℃. Then, the products were dispersed into deionized water (20 mL), followed by the addition of ammonium hydroxide (3.5 mL) and incubation for another 3 h at 98 ℃. The final HMON products were obtained through centrifugation, washing and dried under vacuum.

### Synthesis of HHBP nanoparticles

HMONs (200 mg) were dispersed in the methanol solution and functionalized with 3-aminopropyltriethoxysilane (APTES, 1 mL) for 16 h at 120 ℃. Then, the precipitate was collected by centrifugation, washed with ethanol several times, and finally dried to obtain the HMONs-NH_2_ nanoparticles. Next, the 3BP was anchored onto the surface of HMONs-NH_2_ via the amidation reaction. Briefly, HMONs-NH_2_ (80 mg) nanoparticles were dispersed in DMSO/deionized water mixture solution, containing EDC (12.0 mg) and NHS (7 mg, 0.12 mmoL). The reaction system was stirred for 6 h at room temperature, to which 3BP (10 mg) was slowly added, followed by vigorous stirring for another 12 h. The product (HMONs-3BP) was obtained via centrifugation and purified with ethanol.

For hematoporphyrin monomethyl ether (HMME) loading, HMONs-3BP was dispersed in the HMME methanol solution at different mass ratios (4:1; 2:1; 1:1; 2:3; 2:4) and stirred at room temperature for 24 h. After centrifugation and purification with methanol several times, the HMME-loaded formulations (designated as HMME@HMONs-3BP) were obtained. Meanwhile, the collected supernatant was evaluated for its HMME-loading capacity by UV-vis analysis. Finally, the PEGylated nanoparticles (HHBP) were added to the mPEG-COOH in the HMME@HMONs-3BP dispersion and stirred for 12 h. The final product obtained was washed and re-dispersed in PBS for further use.

### Qualitative and quantitative ^1^O_2_ detection

The generation of ^1^O_2_ was determined by electron spin resonance (ESR; Brooke A300, Germany) with the trapping agent 2,2,6,6-tetramethylpiperidine (TEMP). Typically, HHBP (150 µg/mL) was exposed to US irradiation (1.0 W/cm^2^, 1.0 MHz, 50% duty circle) for 1 min in a mixture containing TEMP (100 mM), and the mixture was immediately subjected to ESR spectroscopy. As control groups, PBS with and without US irradiation and HHBP without US irradiation were also tested.

To quantitatively evaluate the production of ^1^O_2_, HHBP was dispersed in DMF (150 µg/mL, 2.96 mL), followed by the addition of 1,3-diphenylisobenzofuran (DPBF, 8 mM, 40 µL). Then, the mixture was treated with US irradiation (1.0 W/cm^2^, 1.0 MHz, 50% duty circle) for 1 min, and the absorbance intensity was recorded by UV-vis spectrophotometer at the wavelength of 398 nm.

### In vitro **pH/GSH/US-responsive HMME release**

First, the biodegradation behavior of the HHBP nanoparticles in the simulated body fluid (SBF) containing GSH at different concentrations (0, 5, and 10 mM) was evaluated by transmission electron microscopy (TEM) observation. The release behavior of HMME was measured by using a dialysis method through a UV-Vis spectrophotometer. Typically, the HHBP dispersion (1 mg/mL HMME equivalent, 0.5 mL DMSO, and 4.5 mL buffer solution) was placed in a dialysis bag under different incubation-condition groups: (1) PBS, pH 7.4; (2) ABS, pH 5.5; (3) PBS, pH 7.4, GSH (10 mM); and (4) ABS, pH 5.5, GSH (10 mM) at 37 °C under constant stirring. Meanwhile, 0.5 mL of the releasing solution outside the dialysis bag was collected for UV-vis analysis at a 398 nm wavelength to determine the amount of released HMME. In addition, the US-triggered release behavior was assessed under the same condition of group 3 and 4, and then exposed to US irradiation (1.0 W/cm^2^, 1.0 MHz, 50% duty circle) over 1 min at 1, 4, 8, and 16 h of incubation.

### In vitro **ROS production at cellular level**

2’,7’-dichlorodihydrofluorescein diacetate (DCFH-DA) was served as a probe to detect intracellular ROS generation. Briefly, 4T1 cells were incubated into 12-well plates at the density of 1 × 10^5^ cells overnight. Subsequently, the cell medium was replaced and the cells were treated with different formulations including PBS, HMME@HMONs-PEG, and HHBP for another 4 h. Then, the cells were further exposed to US irradiation (1.0 W/cm^2^, 1.0 MHz, 50% duty circle, 1 min) and incubated with DCFH-DA (1:1000 dilution) for 30 min according to the manufacturer instructions. The treated cells were then washed thrice with PBS and imaged by fluorescence microscopy. Finally, the cells were collected and subjected to quantitative analysis by measuring the fluorescence intensity by flow cytometry (FACS-Calibur; BD Biosciences).

### In vitro **nanotherapeutic performance of HHBP**

4T1 cells were seeded into 96-well plates at the density of 1 × 10^4^ cells and treated with PBS, HMME@HMONs-PEG, 3BP-HMONs-PEG or HHBP for 4 h. Then, a part of the cells was further exposed to US irradiation (1.0 W/cm^2^, 1.0 MHz, 50% duty circle, 1 min). After incubation for a total time of 12 h, the cells were washed thrice with PBS and enumerated for cell viability by the CCK-8 assay. Correspondingly, all treated cells were digested with trypsin and stained by using the Calcein-AM/PI kit, according to the manufacturer’s instructions. After incubation for 15 min, the cells were visualized under a fluorescence microscope. Next, the cell apoptosis detection assay was performed to further investigate the cell death mechanism. Briefly, 4T1 cells were seeded into 6-well plates for 24 h and treated as for the CCK-8 assay. Then, the cells were washed with PBS and collected by centrifugation, followed by treatment with the Annexin V-FITC apoptosis detection kit and quantification by flow cytometer.

### Detection of HIF-1α and HK-II by Western blotting

The expression of HK-II and hypoxia inducible factor-1α (HIF-α) in cells was evaluated by Western blotting. The BCA protein concentration determination kit was used to measure the protein concentration, as per the specific method recommended by the manufacturer. Briefly, the 4T1 cells were inoculated into 6-well plates (2 × 10^5^ cells/well) and incubated with PBS, HMME@HMONs-PEG (100 µg/mL), or HHBP (100 µg/mL) for 4 h. Then, a part of the cells was further exposed to US irradiation (1.0 W/cm^2^, 1.0 MHz, 50% duty circle, 1 min). After incubation for a total time of 6 h, a protease inhibitor was added to the cell lysates to extract the proteins, which were then separated by 10% sodium dodecyl sulfate-polyacrylamide gel electrophoresis and then transferred onto the polyvinylidene fluoride membrane (Millipore, IPVH00010). The membrane was decolorized and sealed with 5% skim milk (0.5%TBST) on a shaker for 1 h. The primary antibodies HIF-1α (1:2000 dilution, ab16066; Abcam,) and HK- II (1:5000 dilution, ab227198; Abcam) were diluted and incubated with the membrane overnight at 4 ℃, followed by washing with TBST thrice and hybridizing with the relevant secondary antibody for 1 h. Finally, the film was observed by chemiluminescence. The protein expression and the band strength were quantified using the Image-Pro Plus 6.0 software. β-actin served as the loading control. Each treatment included three parallel samples, and the data were expressed as mean ± standard deviation (n = 3).

### Animals and tumor model

Healthy female BALB/c nude mice (age: 4–6 weeks) were provided by the Animal Center of Shanghai Sixth People’s Hospital. All animal experiments were conducted as per the protocols approved by the Institutional Animal Care and Use Committee (IACUC) of Shanghai Jiao Tong University Affiliated Sixth People’s Hospital (Animal Welfare Ethics acceptance number No: DWLL2020-0582). The 4T1 cell subcutaneous tumor model was established by subcutaneously injecting 100 µL of 2 × 10^6^ cells suspension into the female nude mice. The bodyweight of the mice was monitored and the tumor volume (V) was calculated using the following equation: V = 0.5 L × W^2^. Where, L and W represent the length and width of the tumor, respectively.

### In vivo **antitumor assays**

When the tumor size reached 150 mm^3^, the mice were randomly divided into 5 groups (n = 6 mice in each group), as follows: (I) PBS, (II) HMME@HMONs-PEG, (III) HHBP, (IV) HMME@HMONs-PEG + US, and (V) HHBP + US. The mice were intravenously injected with 100 µL of HMME@HMONs-PEG (HMME: 8 mg/kg) or HHBP (HMME: 8 mg/kg, 3BP: 3.4 mg/kg), respectively. The parameters of US irradiation were set as follows: 1.5 W/cm^2^, 1.0 MHz, 50% duty circle for 5 min. The mice were administrated with the drugs and irradiated on days 1 and 4. After 12 h of treatments, tumor blocks of mice were removed and frozen at –20 °C for DHE staining to detect ROS. HIF-1α staining was performed to measure the extent of tumor hypoxia, and LC3 staining was conducted to detect the autophagy level. After treatments, the tumor-bearing nude mice were sacrificed and their tumor tissues were collected for H&E, Ki67, and TUNEL staining. In addition, the major issues were dissected to prepare paraffin sections for further H&E staining.

### Statistical analysis

Data were expressed as the mean or mean ± standard deviation. The statistical significance was determined by Student’s *t*-test: ns: P > 0.05, *P < 0.05, **P < 0.01, ***P < 0.001. All statistical analyses were conducted using the SPSS software.

## Results and discussion

### Design, fabrication, and characterization of HHBP nanosonosensitizers

**Fig. 2:**
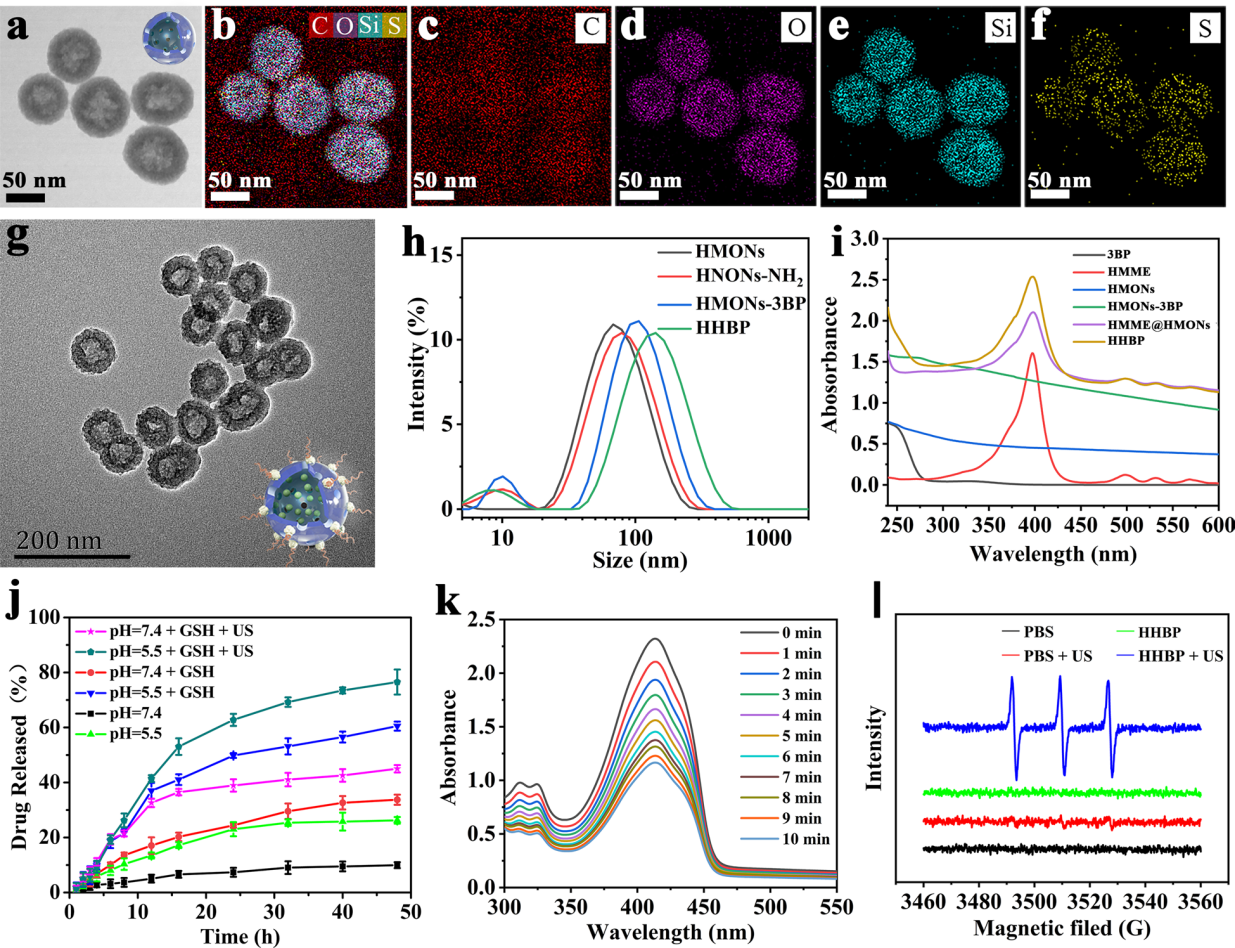
Characterization and in vitro performance of HHBP. **a** TEM image and (**b**–**f**) the elemental mappings of C, O, Si, and S of HMONs. **g** TEM image of HHBP. **h** Hydrodynamic diameter of HMONs, HMONs-NH_2_, HMONs-3BP, and HHBP. **i** The UV-vis absorption spectra of 3BP, HMME, HMONs, HMONs-3BP, HMME@HMONs-PEG, and HHBP. **j** The release profiles of HMME from HHBP at different pHs and GSH concentrations with or without US irradiation (1.0 W/cm^2^, 1.0 MHz, 50% duty circle, 1 min, n = 3). **k** Time-dependent oxidation of DPBF indicating ^1^O_2_ generation by HHBP under US irradiation. **l** ESR spectra demonstrating ^1^O_2_ generation for different groups

The procedure for the fabrication of HHBP nanosonosensitizer and the corresponding therapeutic mechanism is illustrated in Fig. 1. To endow the nanosonosensitizer with TME-responsive biodegradability, we introduced disulfide bond components into the framework of mesoporous silica to prepare organic-inorganic hybrid HMONs for the delivery of both 3BP and HMME. Typically, HMONs were prepared by employing an “ammonia-assisted selective etching” approach reported [38–40]. Initially, the monodispersed SiO_2_@MONs nanoparticles were synthesized through the co-hydrolysis of TEOS and BTDS by employing CTAC as the structural-directing agent [41, 42]. As visualized by TEM observation, the as-synthesized SiO_2_@MONs exhibited a core/shell structure (Additional file 1: Fig. S1). After etching in an ammonia solution, the SiO_2_ core was removed and the hollow HMONs of approximately 85 nm size were obtained (Fig. 2a). Elemental mapping images showed the presence of sulfur with the coexistence of silicon, carbon, and oxygen in HMONs (Fig. 2b–f), suggesting the formation of disulfide bond hybrid silsesquioxane framework within HMONs, as further proved by energy-dispersive spectrometry (EDS) analyses (Additional file 1: Fig. S2). Meanwhile, the characteristic C signals in ^13^C cross-polarization solid-state NMR spectra and silicon resonances in the ^29^Si magic-angle spinning confirmed the successful hybridization of disulfide bond into the framework of HMONs (Additional file 1: Fig. S3). The amino-functionalized HMONs (HMONs-NH_2_) were prepared and then covalently modified with 3BP through an amide reaction between the carboxyl groups of 3BP and the amino groups of HMONs-NH_2_ (HMONs-3BP). The presence of peaks of the amide group at 1623 (C=O stretching vibration) and 3278/cm (N-H stretching vibration [43]) in the Fourier-transformed infrared (FT-IR) spectrum demonstrated the successful formation of HMONs-3BP (Additional file 1: Fig. S4). According to the N_2_ absorption-desorption isotherms, the BET surface area of HMONs decreased from 481 m^2^/g to 301 m^2^/g after the incorporation of 3BP (Additional file 1: Fig. S5a), while the pore size decreased from 4.4 nm to 3.8 nm approximately (Additional file 1: Fig. S5b). Even so, these characterization results above evidently confirmed the porosity and hollow feature of the as-prepared HMONs-3BP, which is remained highly suitable for the encapsulation of hydrophobic molecules. Thus, organic sonosensitizers HMME was next loaded into the hollow cavity of the HMONs-3BP (designated as HMME@HMONs-3BP). The nanosonosensitizer was further PEGylated through noncovalent interactions for improving the stability (yielding HHBP). TEM observations revealed that the surface engineering and drug loading exhibited a negligible change in morphology (Fig. 2g), while the hydrodynamic diameter of HHBP was increased to 142 nm (Fig. 2h), which is slightly larger than that of HMONs-3BP. After loading of HMME and PEGylation, the BET surface area was decreased to 206 m^2^/g and the mesopore size was also dramatically reduced to approximately 2.4 nm (Additional file 1: Fig. S5). Moreover, the serial changes in the zeta potential further reconfirmed the desirable synthesis at each step (Additional file 1: Fig. S6). Furthermore, UV-vis spectra of HHBP depicted the HMME characteristic absorption peaks at 398 nm (Fig. 2i). The loading capacity of HMME was found to be approximately 38% (HMME: HMONs-3BP, w/w) when the HMONs-3BP/ HMME feeding ratio was 0.5 (Additional file 1: Fig. S7). These results suggested that HMONs could be efficiently loaded with HMME and modified with 3BP followed by coating with PEG on the surface. Moreover, HHBP has a good colloid stability and disperse well in PBS, saline and DMEM medium without obvious aggregation, even standing for 7 days. Correspondingly, the absorbance of HHBP at 300–500 nm did not change significantly (Additional file 1: Fig. S8).

As the disulfide-bridged silsesquioxane framework of HMONs can be cleaved in the reductive TME [44, 45], the biodegradation behavior of HHBP was evaluated in the SBF (GSH, 5 mM or 10 mM). Based on the TEM images, HHBP in SBF without GSH showed a certain extent of albeit inconspicuous biodegradation during 1 week of immersion in SBF. In contrast, HHBP was inclined to be gradually degraded in the SBF solution containing 5 mM GSH, demonstrating a time-dependent biodegradable behavior (Additional file 1: Fig. S9). Especially, the biodegradation rate was significantly quickened and the nanoparticles were found to be entirely biodegraded in the SBF solution containing GSH (10 mM) for 7 d. These results indicated the GSH-responsive biodegradability of the HHBP nanosonosensitizers.

### In vitro **HMME release and the SDT effect of HHBP**

Considering the unique GSH-sensitive biodegradation behavior of HHBP, we further evaluated their HMME releasing performance and behavior under different GSH concentrations and pH values (Fig. 2j). As expected, the HMME release behavior from HHBP was highly dependent on the pH values as well as the GSH concentrations. The percentage of HMME released after 24 h was found to be < 10% at pH 7.4 in the absence of GSH, indicating the high stability of HHBP under physiological conditions. In contrast, the amount of released HMME dramatically increased to 26.2% at pH 5.5, which can be attributed to the electrostatic interaction of HMME with the HMONs was reduced at low pH, thus leading to the HMME release [46]. Notably, the drug-releasing percentage sharply increased to ≈ 33.7% and ≈ 60.5% under the respective GSH concentrations of 10 mM at pH 7.4 and 5.5. This finding can be attributed to the gradual biodegradation of the HMONs framework caused by GSH-induced cleavage of the disulfide bond. Interestingly, the release rates were further enhanced and approximately 76.5% HMME release was observed after 24 h upon exposure to US irradiation, which may be attributable to the dissociation of HMME from HMONs caused by the mechanical/cavitation effects of US [2, 47, 48]. Taking into consideration that the tumor environment is distinguished by mild acidic and high GSH concentration, the pH/GSH/US tri-stimuli-responsive HHBP were expected to deliver hydrophobic sonosensitizers for substantially enhanced SDT efficacy.

Subsequently, the SDT performance of HHBP was evaluated based on the presence of HMME, with DPBF as the ^1^O_2_ probe to determine the ROS production under US irradiation (Fig. 2k). After irradiation with the US, the production of ^1^O_2_ was further improved, as evidenced by the attenuated UV absorption peak of DPBF at 398 nm when the US irradiation time increased. In addition, ESR with the spin traps of TEMP were also acquired. According to the ESR spectra (Fig. 2l), the strong ^1^O_2_ signal (1:1:1) was detected in the HHBP + US group, while no obvious ESR signal was observed in the HHBP-alone group and the PBS group, despite irradiation with US (1.0 W/cm^2^, 1.0 MHz, 50% duty circle, 1 min). The above results indicated that HHBP could be an ideal nanosonosensitizer for US-triggered ROS generation.

### **Intracellular uptake and hypoxia alleviation of HHBP**

**Fig. 3:**
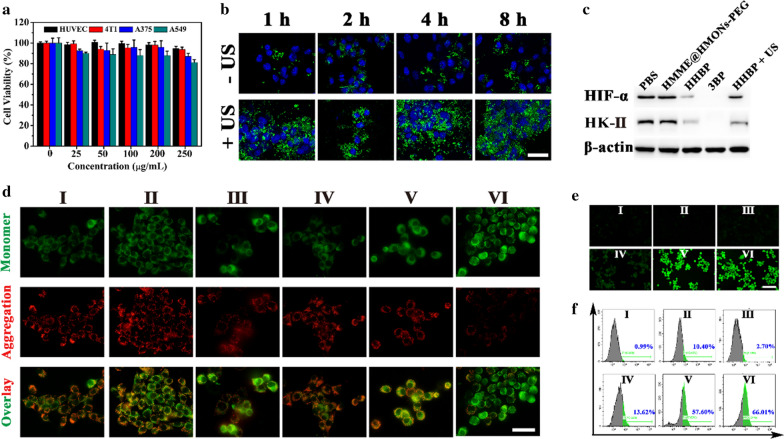
Intracellular uptake, hypoxia alleviation, and SDT efficacy of HHBP. **a** The relative viabilities of HUVEC, 4T1, A375, and A549 cells after incubation with HMONs-PEG nanoparticles (concentrations of 25, 50, 100, 200, and 250 µg/mL) for 24 h. **b** CLSM images of 4T1 cells incubated with FITC-conjugated HHBP for 1, 2, 4, and 8 h without or with US irradiation. Scale bar = 25 μm. **c** Western blotting results of HIF-1α and HK-II expression in 4T1 cells after different treatments. Fluorescence images of 4T1 cells after different treatments and staining with **d** JC-1 dye and **e** an intracellular ROS probe, DCFH-DA. Scale bar = 50 and 100 μm. **f** Flow cytometry data showing intracellular total ROS after different treatments. In panels **d**, **e**, and **f **treatment groups are denoted as: (I) PBS, (II) HMME@HMONs-PEG, (III) HHBP, (IV) PBS + US, (V) HMME@HMONs-PEG + US and (VI) HHBP + US

Inspired by the aforementioned results, we next evaluated the cellular uptake and O_2_-consumption reduction ability of HHBP in 4T1 cells. Initially, the in vitro cytotoxicity of HMONs-PEG was determined by using the CCK-8 assay. HMONs-PEG showed no significant cytotoxicity to various cancer cells in vitro (Fig. 3a), including HUVEC, 4T1, A375, and A549 cells, even at high concentrations of 250 µg/mL, thereby demonstrating the relatively good biocompatibility of HMONs-PEG. In order to evaluate the intracellular endocytosis behavior, the cellular uptake efficiency of HHBP in the cells was investigated. Confocal laser scanning microscopy (CLSM) observations indicated a time-dependent endocytosis process, as evidenced by the increased FITC signals at extended co-incubation durations (1, 2, 4, and 8 h) of HHBP with 4T1 cancer cells (Fig. 3b). More excitingly, after further irradiation with the US, a stronger green fluorescence was detected in 4T1 cells in the presence of HHBP. This phenomenon mainly due to the local cavitation effect induced minor disruptions to cell membrane for improving the cell uptake of nanoparticles [10]. In addition, flow cytometry analyses were also performed to determine the fluorescence intensity of FITC (Additional file 1: Fig. S10). The cells treated with HHBP exhibited a high uptake efficiency with increasing incubation time and the US stimulation could enhance the cellular uptake of nanoparticles which are consistent with the CLSM observation.

As an excellent respiration inhibitor, 3BP is expected to be a hypoxia ameliorator for reducing O_2_ consumption, which is the essential condition for amplifying SDT [49]. To verify this behavior, the expression of HK-II, which involved in the first stage of cellular respiration, was evaluated in the cells after treatment with different formulations by Western blotting (Fig. 3c). Notably, negligible changes in the HK-II and HIF-1α levels were detected in 4T1 cells after incubation with HMME@HMONs-PEG, while HHBP with or without US irradiation could markedly reduce the protein expression levels (Additional file 1: Fig. S11). Arguably, the introduction of 3BP played a vital role in the downregulation of HK-II and HIF-1α. These results prove that the inhibition of HK-II used by such an HHBP exposure could inhibit cellular respiration, which could cause changes in the cellular oxygen consumption pattern. Furthermore, the effect of HHBP under US irradiation on mitochondrial dysfunction was evaluated by measuring the variation in the mitochondrial membrane potential with the commercial JC-1 dye. We found that the 4T1 cells treated with HHBP without or with US irradiation displayed strong green fluorescence (Fig. 3d), which is in marked contrast to the prominent red fluorescence observed after treatment with PBS or HMME@HMONs-PEG, thus indicating that the respiration was further suppressed.

### In vitro **augmented SDT efficacy of engineered HHBP nanosonosensitizer at the cellular level**

**Fig. 4:**
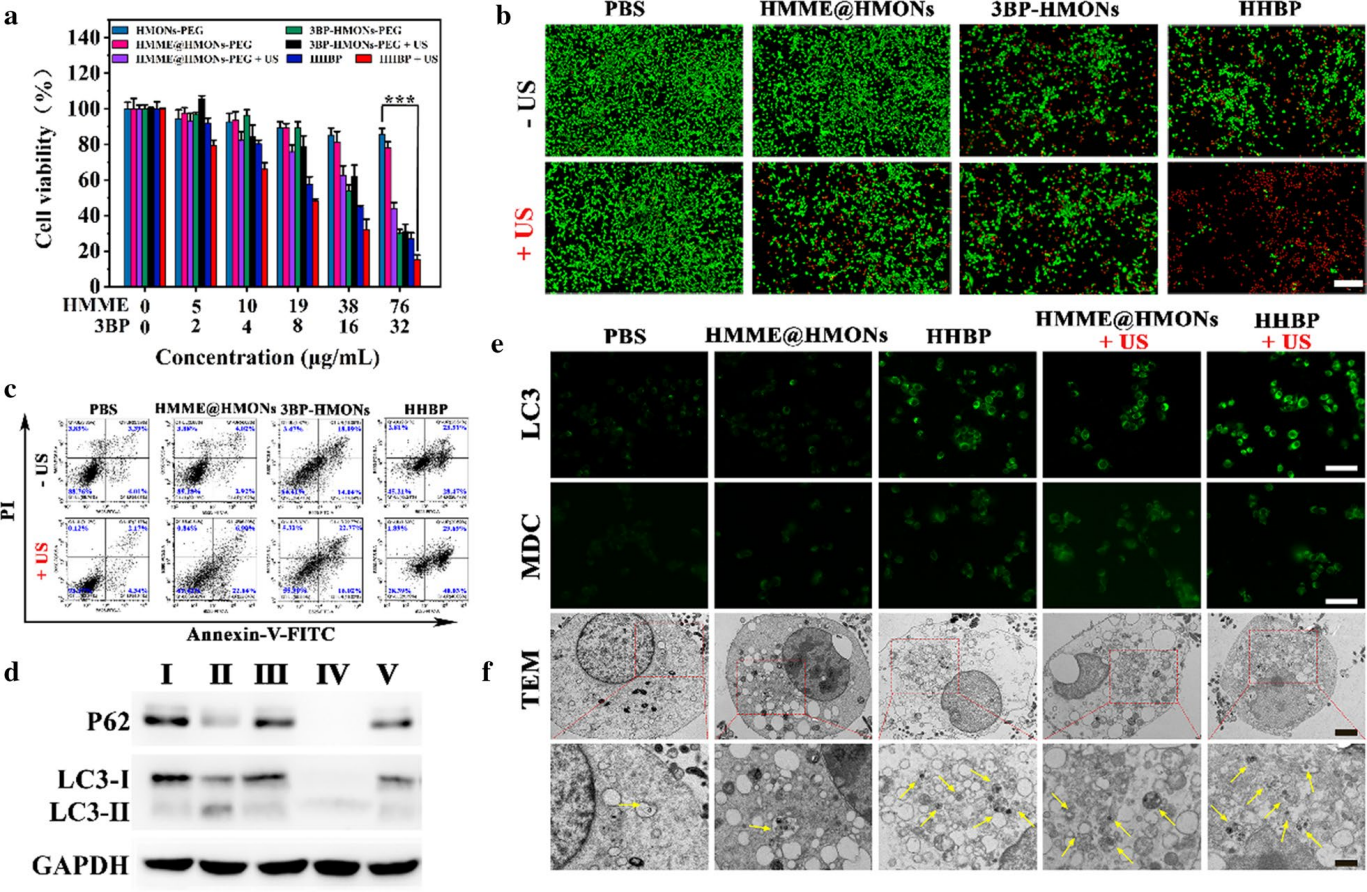
Pro-death autophagy induced by HHBP promoted cancer cell apoptosis. **a** The relative viabilities of 4T1 cells after different treatments, quantified by CCK-8 assay (n = 6). ***P < 0.001. **b** Fluorescence images of 4T1 cells stained by Calcein-AM (green) and PI (red) after treatment with PBS, HMME@HMONs, HHBP without or with US irradiation. Scale bar = 200 μm. **c** Flow cytometry analysis of annexin V-FITC/PI-stained 4T1 cells after treatment with PBS, HMME@HMONs or HHBP without or with US irradiation. **d** Western blotting of the p62, LC3-I, and LC3-II levels in 4T1 cells after different treatments. (I) PBS, (II) HHBP, (III) HMME@HMONs, (IV) HHBP + US, and (V) HMME@HMONs + US. **e** Representative immunofluorescence staining of LC3 punctate dots and MDC-stained fluorescent images after 4T1 cells were treated with HMME@HMONs or HHBP for 4 h with or without US irradiation. Scale bars = 100 μm. **f** TEM images showing the formation of autophagosomes after the 4T1 cells were treated with HMME@HMONs or HHBP for 4 h with or without US irradiation. Scale bars = 2 μm. The yellow arrows in the magnification of TEM images represent the typical structures of autophagosomes. Scale bars = 1 μm

Inspired by the excellent O_2_-economization properties of HHBP nanosonosensitizers, the intracellular ROS generation and in vitro augmented SDT efficacy were further assessed. The intracellular ROS levels were visualized by a of DCFH-DA probe, which could be oxidized into green fluorescent, 2’,7’-dichlorofuorescin (DCF) in the presence of ROS [50, 51]. As revealed in Fig. 3e, negligible fluorescence was detected in the PBS, HMME@HMONs-PEG, and HHBP groups, while the cells treated with the latter two under the US irradiation displayed conspicuous green fluorescence, thus indicating a strong ROS generation. Further quantitative flow cytometric results summarized in Fig. 3f demonstrate that the cellular fluorescence intensity of HHBP + US treatment was significantly higher than that with other treatments. As a result, HHBP could induce plentiful ROS production under US irradiation, displaying a significant potential for SDT against tumors. Encouraged by the prominent ROS generation performance of HHBP, in vitro augmented SDT effect was assessed on 4T1 cells by the CCK-8 assay. As expected, the viabilities of the cells after treatment with HMME@HMONs-PEG + US, 3BP-HMONs-PEG, 3BP-HMONs-PEG + US, HHBP were decreased to 62.5%, 53.8%, 62.2% and 44.9% at the HMME concentration of 38 µg/mL, respectively (Fig. 4a). In contrast, much lower cell viabilities were detected after treatment with HHBP at the corresponding concentrations under US irradiation, suggesting an excellent synergistic effect of SDT and 3BP. Similar results were also revealed by the Calcein-AM/PI stain assay (Fig. 4b). Moreover, the treatment of HHBP under US irradiation could also achieve an effective inhibition effect on diverse tumor cells including A375 and A549 cells with strong concentration-dependent cytotoxicity (Additional file 1: Fig. S12). Significantly, almost no green fluorescence was observed when the cells were treated with HHBP and US irradiation. In addition, the apoptosis effect induced by the synergistic effect was evaluated by the Annexin V-FITC/PI based Flow Cytometer assay (Fig. 4c). The apoptosis/necrosis rate of the cells treated with “HHBP + US” was 69.7%, which was much higher compared with that of other treatments. Taken together, the HHBP nanosonosensitizer could reduce O_2_ consumption for enhanced SDT, thus promising improvement in the therapeutic outcomes of cancer treatment.

### Pro-death autophagy induced by HHBP promoted cancer cell apoptosis

Notably, the in vitro therapeutic outcomes showed that HHBP treatment without US irradiation could also acquire moderate inhibitory effects. Therefore, we believe that 3BP implicates an essential role in inhibiting cell viabilities. In past studies, 3BP can suppress both mitochondrial respiration and glycolysis to result in starvation-induced autophagy [49]. To further verify the hypothesis that 3BP can induce pro-death autophagy, we evaluated the expression of the autophagy-related protein (LC3 and p62) in 4T1 cells receiving different treatments by Western blotting. Notably, the highest cellular autophagy levels were detected after treatment with HHBP plus US irradiation (Fig. 4d), which was proved by p62 inhibition as well as the LC3-II/LC3-I ratio elevation. Quantification results shown in Additional file 1: Fig. S13 further indicate that the LC3-II/LC3-I ratio increased by 98-fold after the HHBP + US treatment when compared with that after the control treatment, while the expression of p62 reduced to 1.5%. These observations demonstrated that the synergistic effect of 3BP and SDT could effectively induce autophagy in 4T1 cells. Furthermore, immunofluorescence staining was performed to characterize the autophagy level by visualized monitoring of the LC3 punctate dots (Fig. 4e). Both HHBP alone and HMME@HMONs-PEG + US treatments exhibited green significant fluorescence signals, which can be attributed to the autophagy induced by 3BP and ROS, respectively. When combined with HHBP and extra US irradiation, the green fluorescence was significantly enhanced. The formation of acidic vesicular autophagosomes during the autophagy process was examined by performing the monodansylcadaverine (MDC) staining (Fig. 4e). The treatment of HHBP incubation plus US exposure resulted in extremely strong green fluorescence of acidic autophagosomes when compared to that of the cells treated with HHBP and HMME@HMONs-PEG + US. To observe the autophagosomes more intuitively, the 4T1 cells receiving different treatments were observed by TEM (Fig. 4f). An unequal quantity of autophagosomes was detected in all treatment groups except for the control group, thus indicating that 3BP or SDT-induced ROS could trigger cell autophagy. Meanwhile, HBBP incubated 4T1 cells exposed by the US represented more autophagic vesicles than those in the other treatments. These results suggested that the synergistic effect can activate excessive autophagy, which in turn promote cell death. Concomitantly, the ATP and lactate level in 4T1 cells were investigated. As shown in Additional file 1: Fig. S14, HMME@HMONs-PEG treatment showed no obvious influence on the intracellular ATP level when compared to that in the control group. However, both 3BP and HHBP dramatically reduced the ATP to 17.8% and 20.2%, respectively, suggesting that the depletion of ATP induced by 3BP could significantly elevate the autophagy level. The data revealed that the content of lactate in the treatment groups of 3BP-contained formulations were significantly lower than that in the control group (Additional file 1: Fig. S15), indicated that 3BP inhibited glycolysis and reduced the production of lactate. These results agree well with the therapeutic evaluations described earlier and offer reliable evidence that the improved anticancer activity of HHBP-mediated augmented SDT could be ascribed to the synergistic effect of apoptosis and autophagy.

### In vivo **biodistribution of the HHBP nanosonosensitizer**

**Fig. 5:**
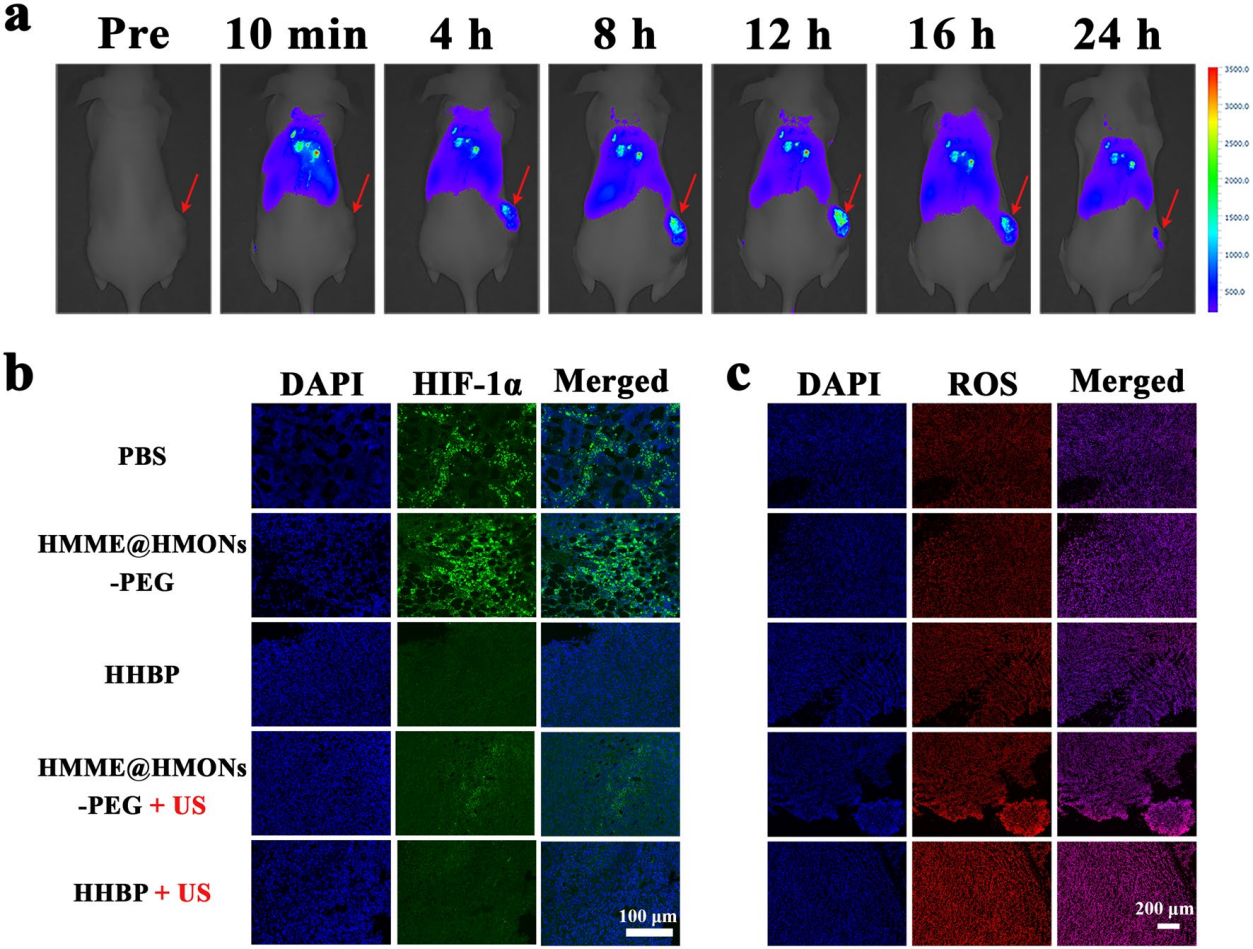
In vivo biodistribution of the HHBP and the alleviation of hypoxia. **a** In vivo fluorescence image of 4T1 tumor-bearing mice at different time points after intravenous injection of ICG-loaded HHBP. The red arrows indicate the tumor sites. **b** HIF-1α staining tumor tissues harvested from 4T1 tumor-bearing mice after different treatments. **c** Fluorescence images of tumor slices were collected at 24 h after different treatments and staining with the ROS probe, dihydroethidium (DHE, red)

The in vitro outstanding properties of the nanosonosensitizer encouraged us to further assess the therapeutic performance on 4T1 breast tumor xenografts in nude mice. For tracking the in vivo distribution and the tumor accumulation behavior of HHBP, a near-infrared dye, indocyanine green (ICG), was loaded to the HHBP through a simple mixing method. From the in vivo fluorescence images (Fig. 5a), the fluorescence signals at the tumor site were increased with increasing time, with the maximum signal detected at 12 h post-injection, which was also maintained at 16 h, followed by a decline due to the degradation of ICG. Correspondingly, the tumor and the main organs of mice were excised at 24 h for ex vivo imaging. Obviously, the ICG-HHBP-treated mice showed strong ICG fluorescence activity at the tumor sites (Additional file 1: Fig. S16). Moreover, a strong fluorescence signal was detected in the liver, which may be attributed to the specific uptake of the mononuclear phagocyte system [52]. These results suggest the favorable tumor accumulation performance of HHBP. Furthermore, the blood circulation profile of the HHBP was evaluated by determining the concentration of Si element in blood collected from the mice at different time points. As shown in Additional file 1: Fig. S17, the pharmacokinetics curve of HHBP followed a classical two-compartment model with a relatively long blood half-life (t_1/2_ =2.09 ± 0.37 h). These results suggested that the obtained nanosonosensitizer features preferable blood circulation stability and has substantial potential for future clinical transformation.

### In vivo **alleviation of hypoxia**

Based on the above-mentioned in vitro findings, HHBP was expected to be a hypoxia modulator for reducing O_2_ consumption, which is deemed beneficial to overcome tumor hypoxia and improve the SDT efficiency. Before testing the antitumor efficacy, the ability of HHBP to ameliorate tumor hypoxia was evaluated by HIF-1α immunofluorescence staining assay. As observed under the fluorescence microscopy (Fig. 5b), extensive green fluorescence was detected in the PBS and HMME@HMONs-PEG groups, thus confirming the overexpression of HIF-1α under hypoxia. In contrast, the indicators of HIF-1α were downregulated after treatment with HHBP with or without US irradiation, which indicated that 3BP could effectively relieve tumor hypoxia.

### In vivo **antitumor efficacy enabled by HHBP nanosonosensitizer**

**Fig. 6:**
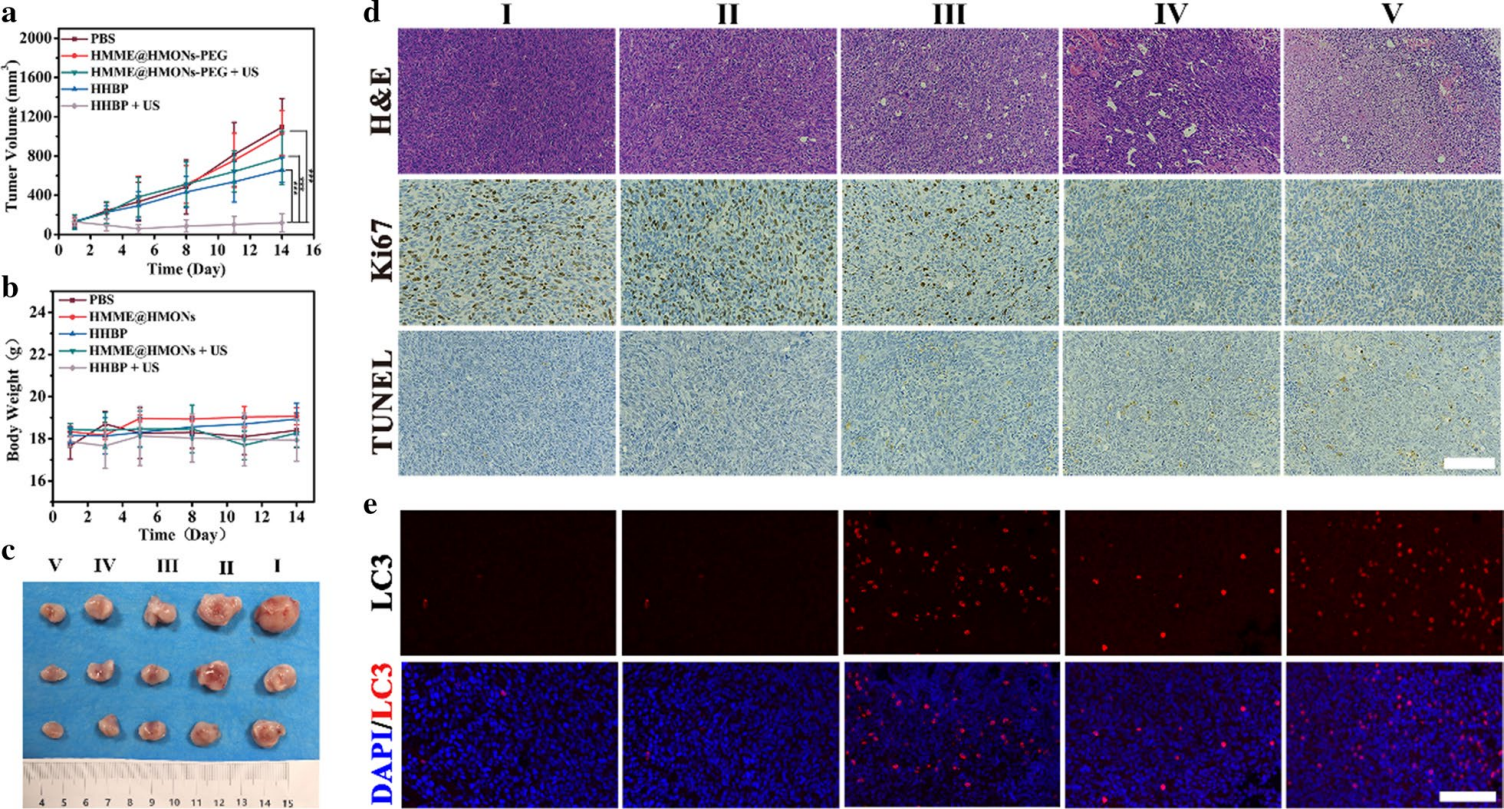
In vivo antitumor efficacy enabled by HHBP. **a** The tumor growth curves following different treatments (n = 3). ***P < 0.001. **b** Body weight of 4T1 tumor-bearing mice after different treatments (n = 3). **c** Representative images of the excised tumors after different treatments. (I) PBS, (II) HMME@HMONs-PEG, (III) HHBP, (IV) HMME@HMONs-PEG + US, and (V) HHBP + US. **d** Histological and immunohistochemical analyses of H&E, Ki67 and TUNEL assays for the excised tumor tissues after different treatments. Scale bar = 200 μm. **e** Representative immunofluorescence images of LC3 in the tumor tissues of mice after different treatments. Scale bar =100 μm

To investigate whether the HHBP-mediated augmented SDT strategy was applied to the in vivo experiments, the therapeutic performance of HHBP against the 4T1 tumor-bearing mice model was performed. Briefly, the mice were randomly divided into 6 groups (n = 5), as follows: (I) PBS, (II) HMME@HMONs-PEG, (III) HMME@HMONs-PEG + US, (IV) HHBP, and (V) HHBP + US. The injected dosage was set to 8 mg/kg HMME and 3.4 mg/kg 3BP (100 µL). The tumor sites of the mice in Group III and V were exposed to US (1.5 W/cm^2^, 1.0 MHz, 50% duty circle) for 5 min and twice as much US exposure on days 1 and 4, respectively. To examine the ROS level at the tumor sites, tumors from different treatment groups were collected after US irradiation for dihydroethidium staining (Fig. 5c). It was observed that both HMME@HMONs-PEG and HHBP could improve the ROS levels under US exposure, and both showed evidently higher levels relative to that with other treatments. More specifically, a higher ROS level could be achieved by HHBP + US treatment, which validated that 3BP can relieve tumor hypoxia and cause tremendous ROS generation through the amplification of the HMME-initiated sonodynamic effect. Following these treatments, the tumor volumes (Fig. 6a) and body weights (Fig. 6b) of the experimental mice were monitored over 2 weeks. We observed rapid tumor growth in the PBS and HMME@HMONs-PEG treated groups, but moderate tumor growth (inhibition rate: 40% and 28.5%, respectively) in the HHBP and HMME@HMONs-PEG plus US treatment groups. More excitingly, the tumors on the mice treated with HHBP and US exposure were found to be most suppressed (inhibition rate: 89.1%). The corresponding images of representative tumors on day 14 in different treatment groups further confirmed that the HHBP + US group realized an intelligent antitumor effect (Fig. 6c). In addition, there was a negligible body-weight loss in mice in all groups during the treatment (Fig. 6b), demonstrating the low adverse effects of all formulations in vivo. The abovementioned desirable therapeutic efficacy may be benefited from the excellent synergistic therapeutic effect, including the 3BP-mediated hypoxia modulation, HMME-based augmented SDT and 3BP-induced excessive activation of autophagy.

To further reveal the performance mechanism of HHBP, the apoptosis and autophagy levels of the collected tumors from all groups were evaluated. Compared to that with other treatments, we detected the most significant large-area histological damaged regions from the H&E staining, the most significant inhibition of cell proliferation from Ki-67 staining, and the highest evident apoptosis of tumor cells from TUNEL staining in the “HHBP + US” treatment group (Fig. 6d). These results are consistent with the foregoing results of in vivo anticancer experiments. Subsequently, we also examined the autophagy levels in tumor sections after different treatments by immunofluorescence imaging staining of LC3 (Fig. 6e). We found that both HMME@HMONs-PEG + US and HHBP alone could improve autophagy levels, according to the green fluorescence of LC3 puncta displayed in the immunofluorescence images. Consistent with the in vitro data, the HHBP group demonstrated the strongest green fluorescence after US irradiation, which represents an excessive activation of autophagy by 3BP and SDT.

Finally, in terms of the safety assessment of HHBP on mice, the H&E sections of the collected major organs did not show any obvious inflammation after injection with HHBP for 3, 7, and 14 days (Additional file 1: Fig. S18), indicating the favorable biocompatibility of HHBP. Concomitantly, the blood sample was collected from healthy female Balb/c mice at the different time points post-injection of HHBP for blood biochemistry and blood routine examinations. No obvious changes were detected concerning the blood routine indexes and liver/kidney function associated biomarker induced by HHBP relative to those in the control group (Additional file 1: Fig. S19), which hints at ignorable systemic toxicity. These results indicated that the fabricated HHBP may augment SDT efficacy with only relatively low systematic toxicity under in vivo conditions.

## Conclusions

In summary, we have proposed an O_2_-economizer strategy to reverse the tumor hypoxia for improving the therapeutic efficacy of SDT, as a substitute for the current mainstream hypoxia-regulation precept of elevating intratumoral O_2_ level. To validate the concept, a biodegradable reduced expenditure bioreactor (HHBP) was constructed through the integration of 3BP and HMME into biodegradable HMONs, which respond to TME and subsequently augments SDT efficacy. The 3BP inhibits cell respiration through downregulation of HK-II expression, which in turn decreases the O_2_ consumption and spares more O_2_, thereby facilitating the SDT by HMME against hypoxic tumors. Importantly, the enhanced ROS generation and 3BP can transform the actor of autophagy from self-protection to pro-death, which is indicated by the increase in the LC3 level and the inhibition of p62 expression. Consequently, HHBP nanosonosensitizers offer better efficient antitumor performance relative to that of the traditional SDT sonosensitizer, which has been systematically verified both in vitro and in vivo. This O_2_-economizer concept not only offers a general strategy to alleviate the tumor hypoxia in SDT but also paves a new way for manipulating autophagic processes to enhance the efficacy of ROS-mediated cancer therapy.

## Supplementary Information

**Additional file 1:** **Fig. S1.** TEM images of SiO_2_@MONs nanoparticles. **Fig. S2.** EDS spectrum of the prepared HMONs. **Fig. S3.** ^13^C and (b) ^29^Si NMR spectra of HMONs. **Fig. S4.** FT-IR spectra of 3BP, HMME, and different HMONs-based nanoparticles. **Fig. S5.** N_2_ adsorption-desorption isotherm and pore-size distribution of HMONs, HMONs-NH_2_, HMME@HMONs-PEG, and HHBP nanoparticles. **Fig. S6.** zeta potentials of HMONs, HMONs-NH_2_, HMME@HMONs-PEG, and HHBP aqueous solution. **Fig. S7.** HMME loading capacities on HMONs-3BP-PEG NPs (w/w%) with different HMONs-3BP/HMME feeding ratios. **Fig S8.** Stability of HHBP in different solutions. **Fig. S9.** TEM images of HHBP dispersed in SBF at varied GSH concentrations (0, 5, and 10 mM) for 1, 5, and 7 days. **Fig. S10.** Flow cytometry analyses of 4T1 cells incubated with FITC-conjugated HHBP. **Fig. S11.** Quantitative determination of the relative HIF-1α and HK-II expression from Western blotting results. **Fig. S12.** Relative viabilities of A549 and A375 cells after different treatments. **Fig. S13.** Quantitative analysis of LC3-II/LC3-I and relative p62 expression after different treatments from Western blotting results. **Fig. S14.** ATP levels in 4T1 cells after different treatments. **Fig. S15.** Intracellular lactic acid content of 4T1 cells after incubation with different nanoparticles. **Fig. S16.** Ex vivo fluorescence imaging of the tumor and the major organs collected from the mice at 24 h post-injection. **Fig. S17.** Blood circulation lifetime of HHBP after intravenous injection into mice. **Fig. S18.** H&E-stained histological images of major organs from mice treated with PBS versus HHBP. **Fig. S19.** Blood biochemistry and hematology data of Balb/c mice treated with HHBP at different time point after i.v. injection. 

## Data Availability

The datasets and materials used in the study are available from the corresponding author.
