# Correction: Biodegradable reduce expenditure bioreactor for augmented sonodynamic therapy via regulating tumor hypoxia and inducing pro‑death autophagy

**DOI:** 10.1186/s12951-022-01716-y

**Published:** 2022-12-09

**Authors:** Weijuan Zou, Junnian Hao, Jianrong Wu, Xiaojun Cai, Bing Hu, Zhigang Wang, Yuanyi Zheng

**Affiliations:** 1 Department of Ultrasound in Medicine, Shanghai Institute of Ultrasound in Medicine, Shanghai Jiao Tong University Affiliated Sixth People’s Hospital, Shanghai, 200233 People’s Republic of China; 2 Chongqing Key Laboratory of Ultrasound Molecular Imaging, Ultrasound Department of the Second Affiliated Hospital of Chongqing Medical University, Chongqing, 400010 People’s Republic of China; 3 State Key Laboratory of Oncogenes and Related Genes, School of Medicine, Shanghai Jiao Tong University, Shanghai, 200233 People’s Republic of China

**Correction: Journal of Nanobiotechnology (2021) 19: 418** https://doi.org/10.1186/s12951-021-01166-y

Following publication of the original article [1], the authors identified an error in Fig. 6d.

**Fig. 6:**
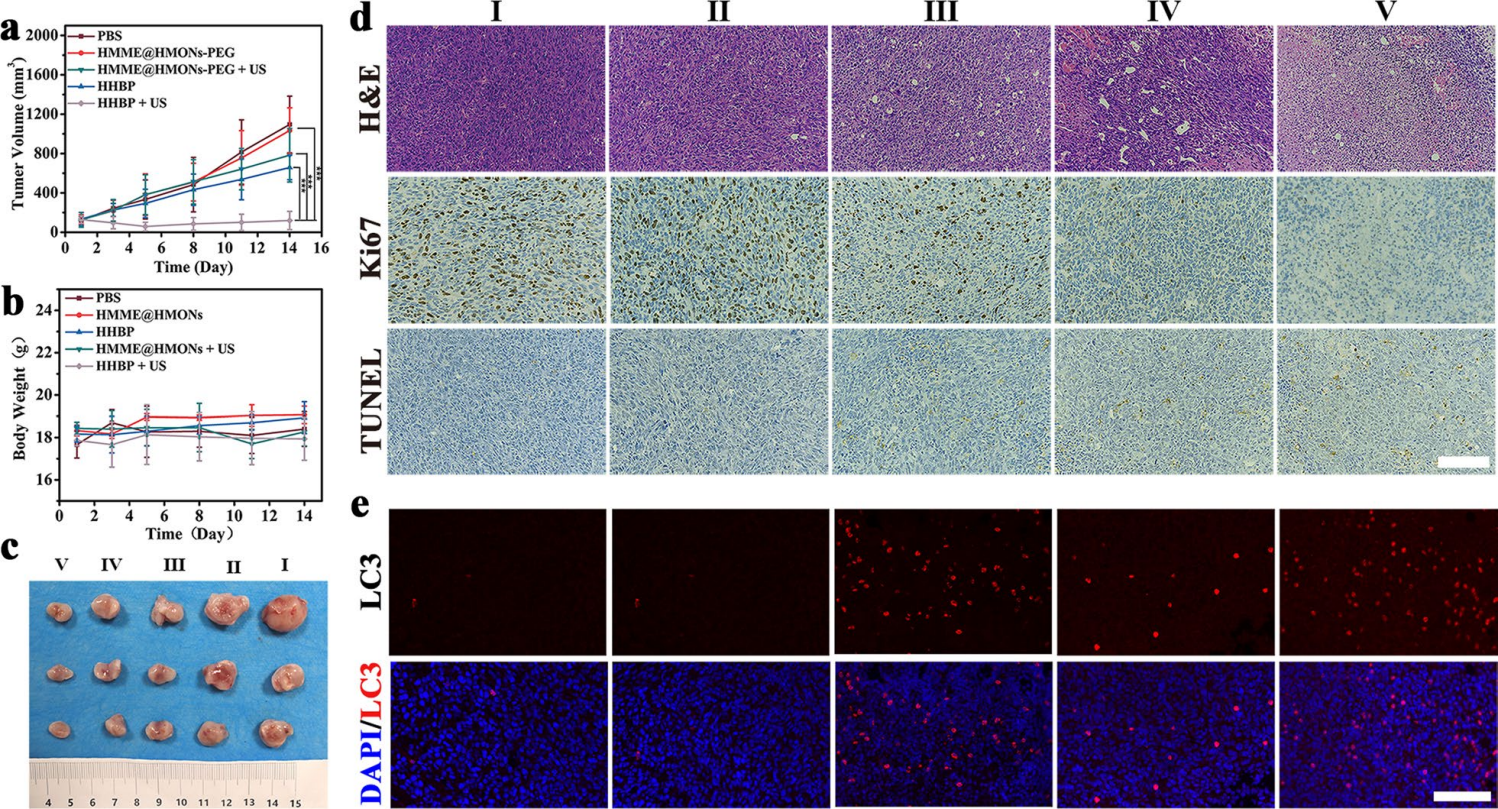
In vivo antitumor efcacy enabled by HHBP. **a** The tumor growth curves following diferent treatments (n = 3). ***P < 0.001. **b** Body weight of 4T1 tumor-bearing mice after diferent treatments (n = 3). **c** Representative images of the excised tumors after diferent treatments. (I) PBS, (II) HMME@HMONs-PEG, (III) HHBP, (IV) HMME@HMONs-PEG + US, and (V) HHBP + US. **d** Histological and immunohistochemical analyses of H&E, Ki67 and TUNEL assays for the excised tumor tissues after diferent treatments. Scale bar = 200 μm. e Representative immunofuorescence images of LC3 in the tumor tissues of mice after diferent treatments. Scale bar = 100 μm

During the process of using integration images tool to permutate and combine multiple data images into one image, the authors inadvertently copied the image from one layer to another and selected the wrong layer, resulting in group IV and V in Ki-67 staining images being cropped from the same image. The correct figure is provided below and the original article has been updated.
