# Supplementary material for: Biodegradable reduce expenditure bioreactor for augmented sonodynamic therapy via regulating tumor hypoxia and inducing pro-death autophagy
Source: J Nanobiotechnology. 2021 Dec 13;19:418. doi: 10.1186/s12951-021-01166-y (PMC8670251; doi:10.1186/s12951-021-01166-y)
Supplement: Supplementary file 1 — Additional file 1: Fig. S1. TEM images of SiO2@MONs nanoparticles. Fig. S2. EDS spectrum of the prepared HMONs. Fig. S3. 13C and (b) 29Si NMR spectra of HMONs. Fig. S4. FT-IR spectra of 3BP, HMME, and different HMONs-based nanoparticles. Fig. S5. N2 adsorption-desorption isotherm and pore-size distribution of HMONs, HMONs-NH2, HMME@HMONs-PEG, and HHBP nanoparticles. Fig. S6. zeta potentials of HMONs, HMONs-NH2, HMME@HMONs-PEG, and HHBP aqueous solution. Fig. S7. HMME loading capacities on HMONs-3BP-PEG NPs (w/w%) with different HMONs-3BP/HMME feeding ratios. Fig S8. Stability of HHBP in different solutions. Fig. S9. TEM images of HHBP dispersed in SBF at varied GSH concentrations (0, 5, and 10 mM) for 1, 5, and 7 days. Fig. S10. Flow cytometry analyses of 4T1 cells incubated with FITC-conjugated HHBP. Fig. S11. Quantitative determination of the relative HIF-1α and HK-II expression from Western blotting results. Fig. S12. Relative viabilities of A549 and A375 cells after different treatments. Fig. S13. Quantitative analysis of LC3-II/LC3-I and relative p62 expression after different treatments from Western blotting results. Fig. S14. ATP levels in 4T1 cells after different treatments. Fig. S15. Intracellular lactic acid content of 4T1 cells after incubation with different nanoparticles. Fig. S16. Ex vivo fluorescence imaging of the tumor and the major organs collected from the mice at 24 h post-injection. Fig. S17. Blood circulation lifetime of HHBP after intravenous injection into mice. Fig. S18. H&E-stained histological images of major organs from mice treated with PBS versus HHBP. Fig. S19. Blood biochemistry and hematology data of Balb/c mice treated with HHBP at different time point after i.v. injection. [file 12951_2021_1166_MOESM1_ESM.docx]

Additional file 1

**Biodegradable reduce expenditure bioreactor for augmented sonodynamic therapy via regulating tumor hypoxia and inducing pro-death autophagy**

Weijuan Zou^1,2†^, Junnian Hao^1†^, Jianrong Wu^1*^ , Xiaojun Cai^1^, Bing Hu^1^, Zhigang Wang^2^, and Yuanyi Zheng^1,2,3*^

* Correspondence: wujr_028@126.com, [zhengyuanyi@sjtu.edu.cn](mailto:zhengyuanyi@sjtu.edu.cn)

^†^Weijuan Zou and Junnian Hao contributed equally to this work

^1^ Department of Ultrasound in Medicine, Shanghai Institute of Ultrasound in Medicine, Shanghai Jiao Tong University Affiliated Sixth People's Hospital, Shanghai 200233, P. R. China

^2^ Chongqing Key Laboratory of Ultrasound Molecular Imaging, Ultrasound Department of the Second Affiliated Hospital of Chongqing Medical University, Chongqing 400010, P. R. China

^3^ State Key Laboratory of Oncogenes and Related Genes, School of Medicine, Shanghai Jiao Tong University, Shanghai 200233, P. R. China

**Additional details on experimental section**

**Materials**

3-bromopyruvic acid (3BP), cetyltrimethylammonium chloride (CTAC) and 3-aminopropyltriethoxysilane (APTES) were purchased from the Aladdin Reagent Co. (Shanghai, China). Hematoporphyrin monomethyl ether (HMME) was provided by Yuanye Biological Technology Co. (Shanghai, China). 1,3-diphenylisobenzofuran (DPBF), N-hydroxy succinimide (NHS) and 1-ethyl-3-(3-dimethylaminopropyl) carbodiimide (EDC) were obtained from Sigma-Aldrichh (MO, USA). Triethanolamine (TEA), tetraethyl orthosilicate (TEOS) and bis(3-triethoxysilylpropyl) disulfide (BTDS) were purchased from Macklin Reagent Co. (Shanghai, China). Glutathione (GSH), fluorescein isothiocyanate (FITC) and MDC staining assay kit were purchased from Solarbio. (Beijing, China). Cell counting kit-8 (CCK-8), 2, 2, 6, 6-tetramethylpiperidine oxide (TEMP), Calcein-AM/PI cell double dyeing kit (Dojindo, C542), mitochondrial membrane potential detection kit JC-1 (JC-1) and DAPGreen-autophgy were obtained from Dojindo Laboratories (Japan). Reactive oxygen species assay kit, ATP luminescence assay kit and Annexin V-FITC apoptosis detection kit were provided by Beyotime Biotechnology Co. (Shanghai, China). LA assay kit was purchased from Solarbio (Beijing, BC2235). Hematoxylin and eosin (H&E), Ki67, dUTP nick-end labeling (TUNEL) and dihydroethidium (DHE) were obtained from Servicebio Co. (Hubei, China). LC3 and HIF-1α primary antibody were provided by Abcam Trading Co. (Shanghai, China).

**Characterization**

The structure and morphology of nanoparticles were characterized by transmission electron microscope (TEM, JEOL JEM 2100F). The fourier transform infrared (FTIR) spectra were measured on a Perkin Elmer Frontier (Perkin Elmer Frontier, USA). Malvern Zetasizer Nanoseries (Malvern, UK) was used to measure the zeta potential and size distributions. The pore size and specific surface area were recorded by the automatic specific surface and porosity analyzer (BET, McASAP2460, USA). UV-vis-NIR absorption spectra were recorded on a UV-vis-NIR spectrophotometer (Perkin Elmer, USA). The HMONs was placed in GSH solution (10 mM) for different time and the changes of structure and morphology were observed by TEM.

**Cell lines and cell culture**

HUVECs (human umbilical vein endothelial cells), 4T1 cells (murine breast cancer cells), A375 cells (Human malignant melanoma cells), A549 cells (Human adenocarcinoma alveolar basal epithelial cells) were purchased from Cell Resource Center (Institute of Life Sciences, Chinese Academy of Sciences). Cells were cultured in DMEM high glucose medium supplemented with 10% FBS and 0.1% penicillin–streptomycin at 37 °C, in a humidified 5% CO_2_ atmosphere.

***In vitro* cell uptake of HHBP**

To obtain FITC-conjugated HHBP, HHBP (60 mg) and FITC (10 mg) was dispersed in 20 mL of ethanol. The mixtures were then stirred overnight at room temperature. The products were obtained by centrifugation, washed with ethanol and redispersed with PBS for further use.

4T1 cells were seeded into 12-well plates (1 × 10^5^ cells/well) and incubated with FITC labeled HHBP (150 µg/mL) for 1, 2, 4 and 8 h. The cells were treated by with or without the US irradiation (1.0 W/cm^2^, 1.0 MHz, 50% duty circle, 1 min), respectively. Then, the cells were washed thrice with PBS, followed by fixing with 4% paraformaldehyde for 15 min. The cell nucleus wwere stained by DAPI for 5 min. All the fluorescence images were acquired by laser scanning confocal microscopy (CLSM, Nikon, Japan). Correspondingly, the quantitative analyses of intracellular fluorescence of FITC was further measured by flow cytometry (BD Accuri C6).

***In vitro* cytotoxicity assay**

The biosafety of HMONs-PEG was evaluated through detecting the viability of different cells, including HUVECs, 4T1 cells, A375 cells and A549 cells by a CCK-8 assay. After incubated with different concentrations of HMONs-PEG for 24 h, the cells were washed thrice with PBS, and then added into the medium (100 μL) containing CCK-8 reagent (10 μL) for another 2 h incubation. Finally, the absorbance at 450 nm was detected by a microplate analyzer.

**Detection of cellular mitochondrial membrane potential**

Mitochondrial embrane potential detection kit (JC-1) was used as a probe to detect the changes of intracellular mitochondrial membrane potential. Briefly, 4T1 cells with corresponding treatments (as mentioned in section of “*In Vitro* Nanotherapeutic Performance of HHBP”) were collected and then stained with JC-1 (1:5000 dilution) for 45 min according to the specification. After that, the cells were washed thrice with PBS and the fluorescence images were acquired by fluorescence microscope.

**Detection of** **intracellular** **autophagy**

For the Western blotting, 4T1 cells with respective treatments were lysed and the protein was extracted. The expression of P62 and LC3 in the cells after different treatments were measured by Western blotting with the protocols as mentioned in section of “Detection of HK-Ⅱ and HIF-1α by Western Blotting”.

MDC and DAPGreen-Autophgy were used to observe intracellular autophagy. 4T1 cells seeded into confocal petri dish (1 × 10^5^ cells/well) and treated according to the manufacturer's instructions in the CCK-8 assay. After that, the cells were stained with MDC or DAPGreen-autophgy according to the specification, and then washed thrice with PBS. The fluorescence images indicated the LC3 dots from different treatments were acquired by fluorescence microscope.

Next, TEM was used to observe intracellular autophagosomes. 4T1 cells with respective treatments were scraped off with a cell scraper and centrifuged. Then,the cells were collected and fixed with glutaraldehyde solution (2.5%) for 1 h. After that, the fixed solution was poured out and the sample was rinsed thrice with PBS (pH 7.0). After fixing with 1% osmium acid solution for another 2 h, the autophagosomes in these cell sections were observed by TEM after dehydration, embedding, secting and staining.

**Detection of** **intracellular ATP level**

ATP luminescence assay kit was used to determinate the ATP level . 4T1 cells seeded into 6-well plates (2 × 10^5^ cells/well) and cultured overnight. The cells were treated as the procedure as-mentioned in the CCK-8 assay. Then, the cells were harvested and the levels of intracellular ATP were measured by an ATP luminescence assay kit according to the specifications. The luminescent signals were measured by a luminometer.

**Detection of intracellular** **lactate level**

LA assay kit was used to determinate the lactate level after different treatments. 4T1 cells were inoculated into 6-well plates (2 × 10^5^ cells/well) and cultured overnight. The cells were treated as the procedure as-mentioned in the CCK-8 assay. Then, the cells were harvested and the levels of intracellular lactate were measured by LA assay kit according to the specifications. The luminescent signals were measured by a luminometer.

***In vivo* fluorescence imaging**

ICG-loaded HHBP nanoparticles were prepared to evaluate the biodistribution of HHBP. 4T1 tumor bearing nude mice were intravenously injected with ICG-loaded HHBP nanoparticles (1.85 mg/kg equivalent ICG, 100 μL). At time points of 0, 0.5, 4, 8, 12, 16, and 24 h post-injection, the fluorescence imaging was conducted at by using a *in vivo* imaging system (VISQUE, invivo Elite). Then, the mice were sacrificed, and the major organs (e.g., heart, liver, spleen, lung, and kidney) and tumors were collected for *ex vivo* imaging under the same conditions.

***In vivo* blood circulation**

At time points of 5, 10, 15, 30 min, 1, 2, 4, 8, and 24 h post-injection of HHBP(HMME: 8 mg/kg, 3BP: 3.4 mg/kg), 10 μL of blood were taken from the tail vein and diluted with aqua regia.Then, the blood circulation profile of the HHBP was evaluated using ICP-AES (PerkinElmer, 2100) by determining the concentration of Si element in blood collected from the mice at different time points.

***In vivo* toxicity evaluation**

The healthy female Balb/c mice were euthanized at 3th day, 7th day, and 14th day (six mice at each group) after injected with 100 μL of HHBP (HMME:8 mg/kg, 3BP: 3.4 mg/kg). In addition, untreated Balb/c mice were served as control group. Blood samples were collected for routine blood analyses and serum biochemical tests.


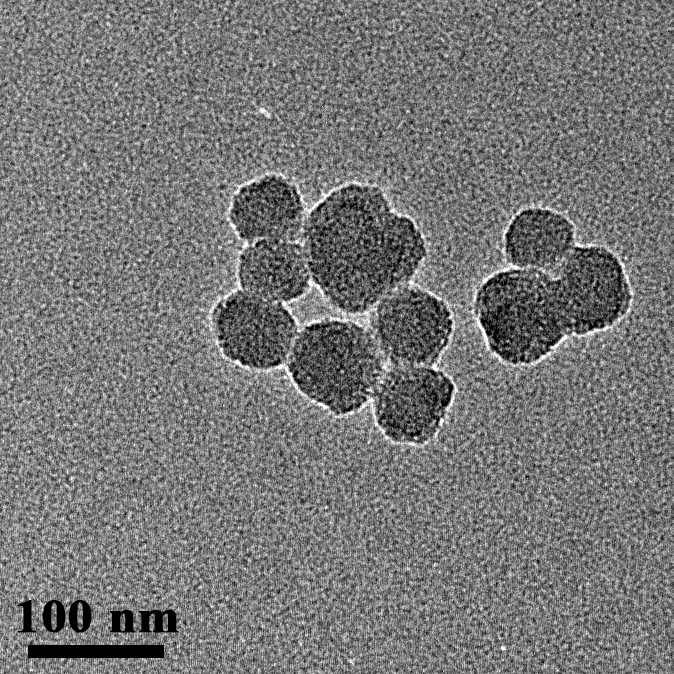


**Fig. S1.** TEM images of SiO_2_@MONs nanoparticles.


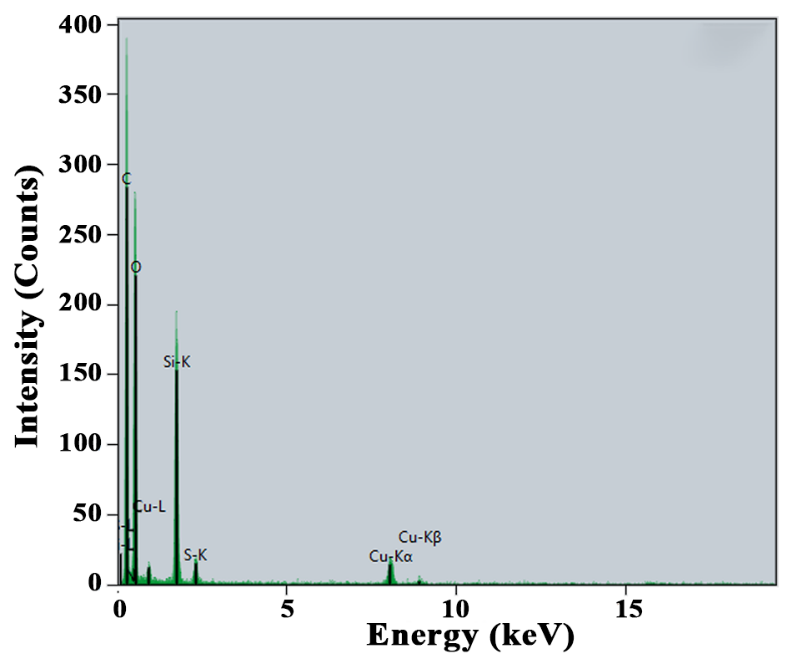


**Fig. S2.** EDS spectrum of the prepared HMONs.


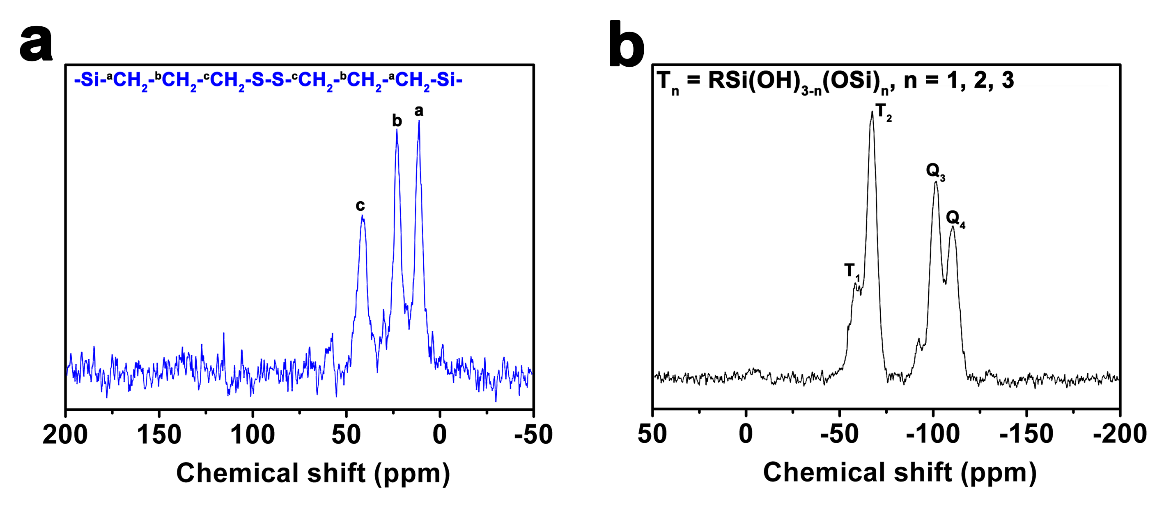


**Fig. S3. a** ^13^C and **b** ^29^Si NMR spectra of HMONs nanoparticles.


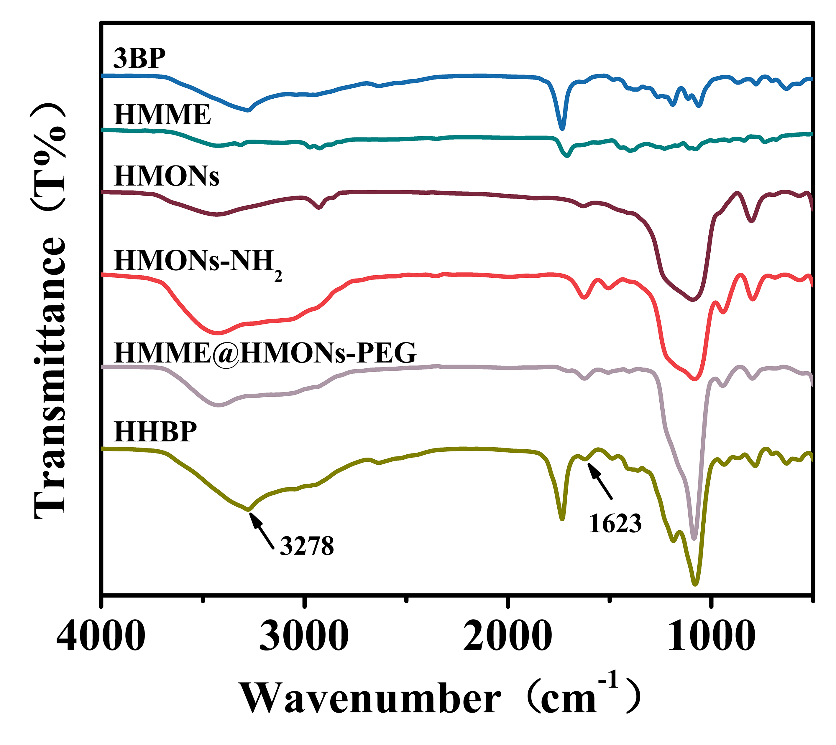


**Fig. S4.** FT-IR spectra of 3BP, HMME, and different HMONs-based nanoparticles.


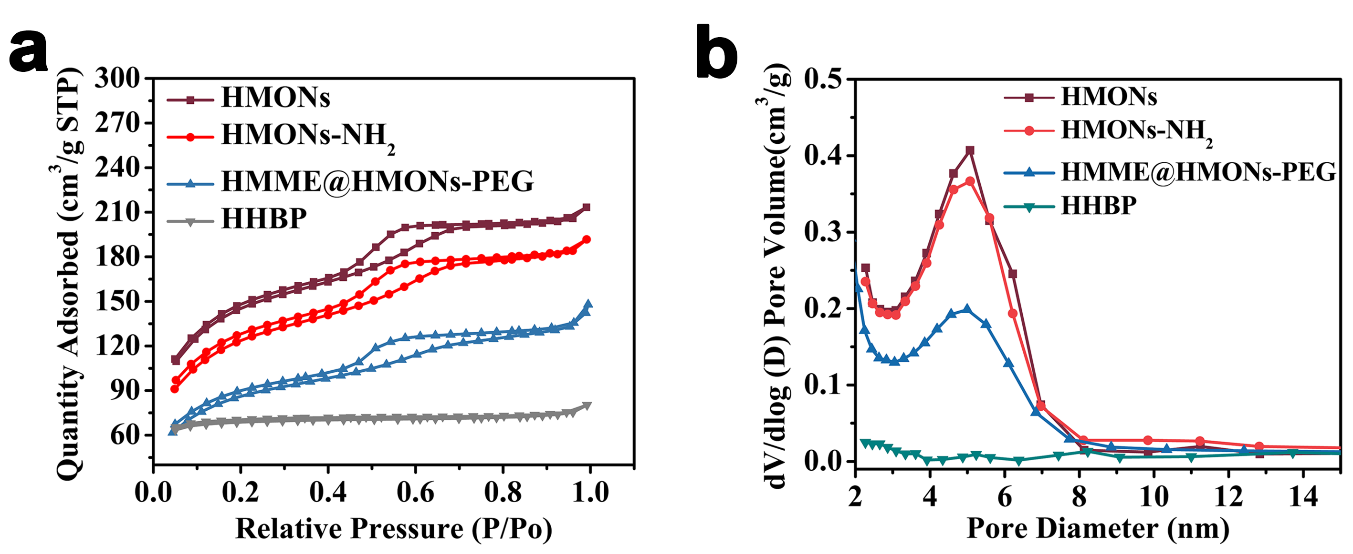


**Fig. S5.** N_2_ adsorption-desorption isotherm **a** and pore-size distribution **b** of HMONs, HMONs-NH_2_, HMME@HMONs-PEG, and HHBP nanoparticles.


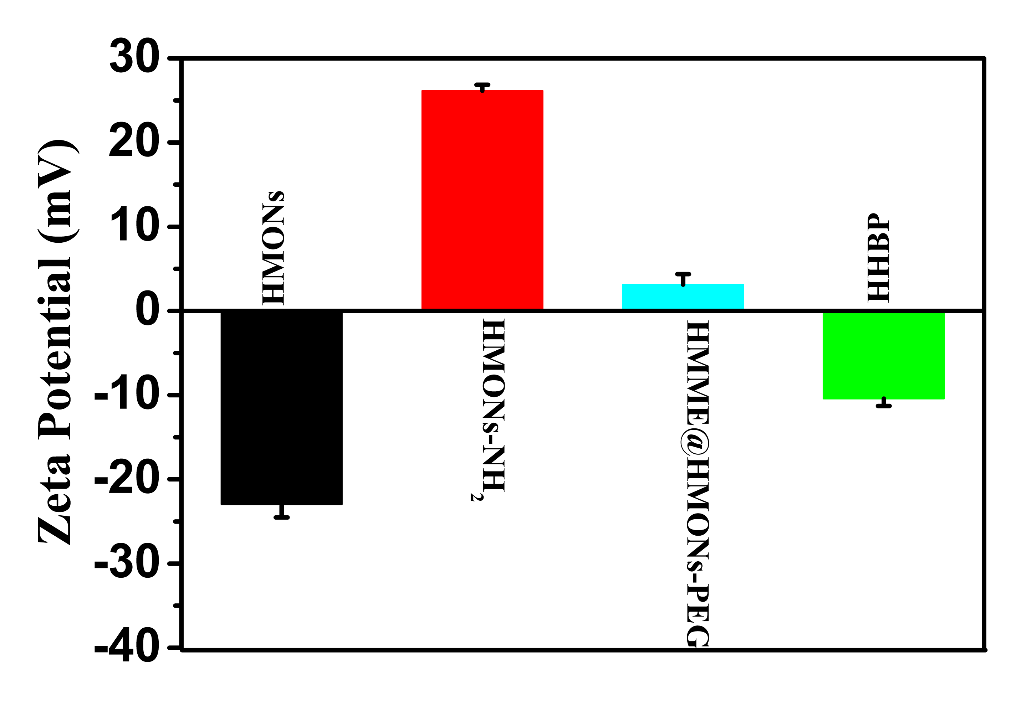


**Fig. S6.** zeta potentials of HMONs, HMONs-NH_2_, HMME@HMONs-PEG, and HHBP aqueous solution after fresh preparation.


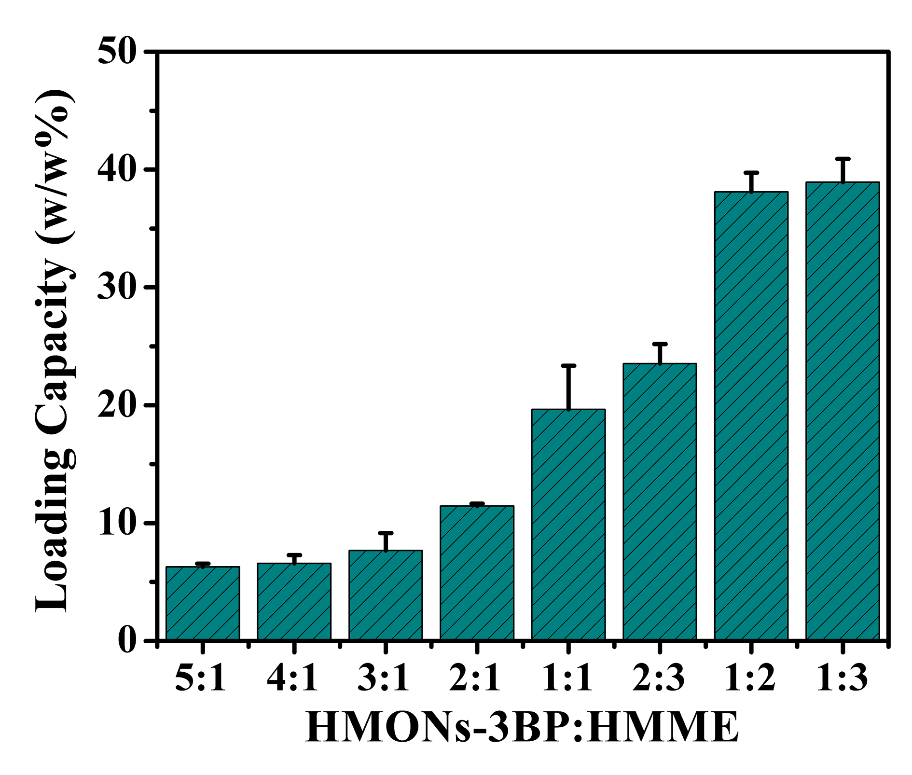


**Fig. S7.** HMME loading capacities on HMONs-3BP-PEG nanoparticles (w/w%) with diffrent HMONs-3BP/HMME feeding ratios (n = 3).


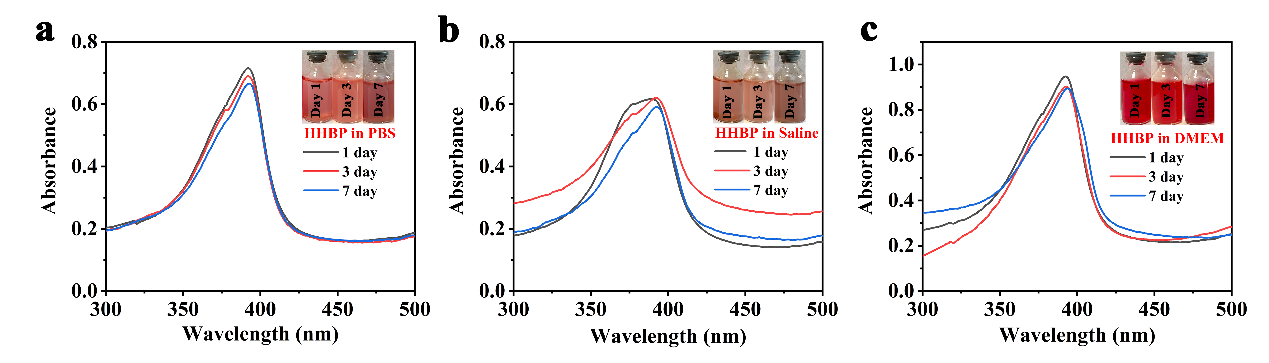


**Fig S8**. Stability of HHBP in different mediums for 1, 3, and 7 days. (a) PBS; (b) saline and (c) DMEM medium. Inset: the photograph of different solutions mixed with HHBP.


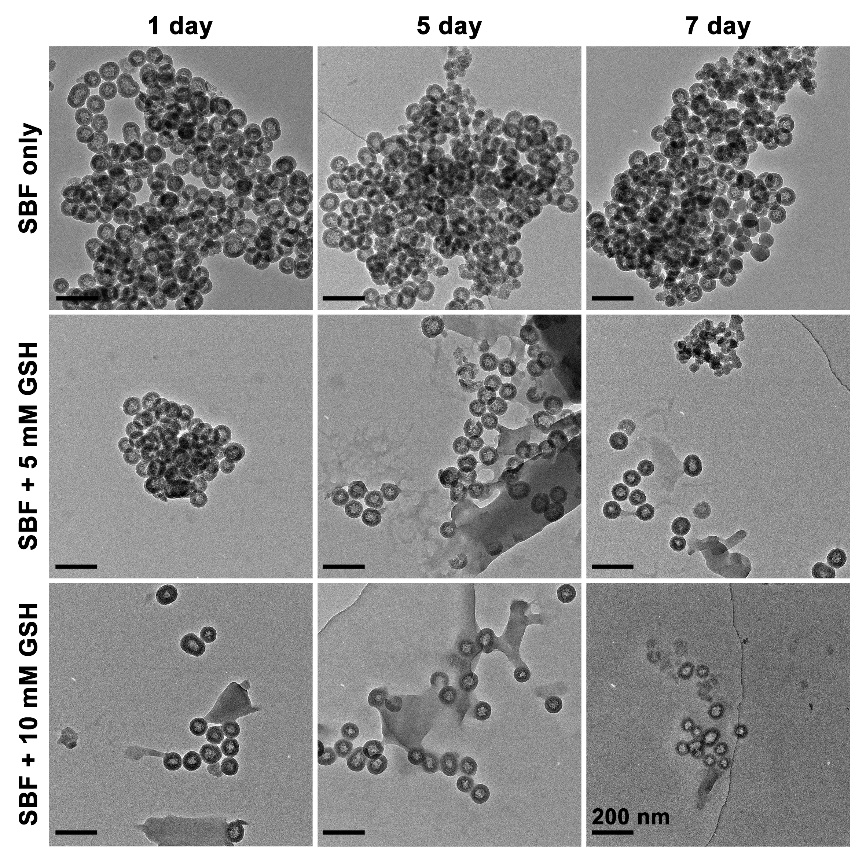


**Fig. S9.** TEM images of HHBP dispersed in SBF at varied GSH concentrations (0, 5, and 10 mM) for 1, 5, and 7 days.


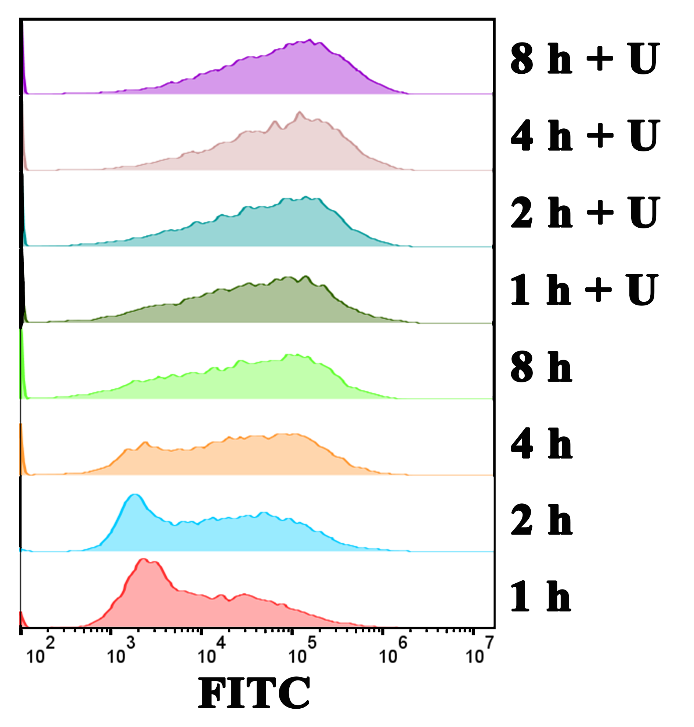


**Fig. S10.** Flow cytometry analyses of 4T1 cells incubated with FITC-conjugated HHBP for 1, 2, 4, and 8 h without or with US irradiation.


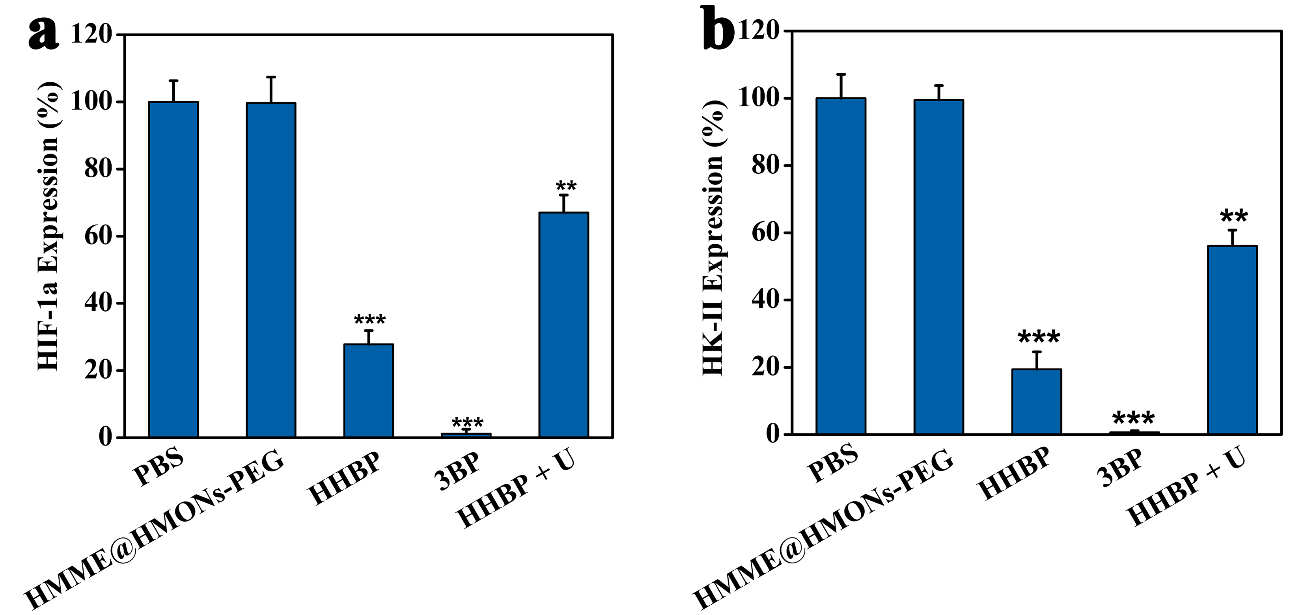


**Fig. S11.** Quantitative determination of the relative HIF-1α **a** and HK-Ⅱ **b** expression from Western blotting results (see **Fig. 2c**). β-Actin was used as a loading control was used as a loading control. **P < 0.01, ***P < 0.001. Error bars indicate standard deviation (n = 3).


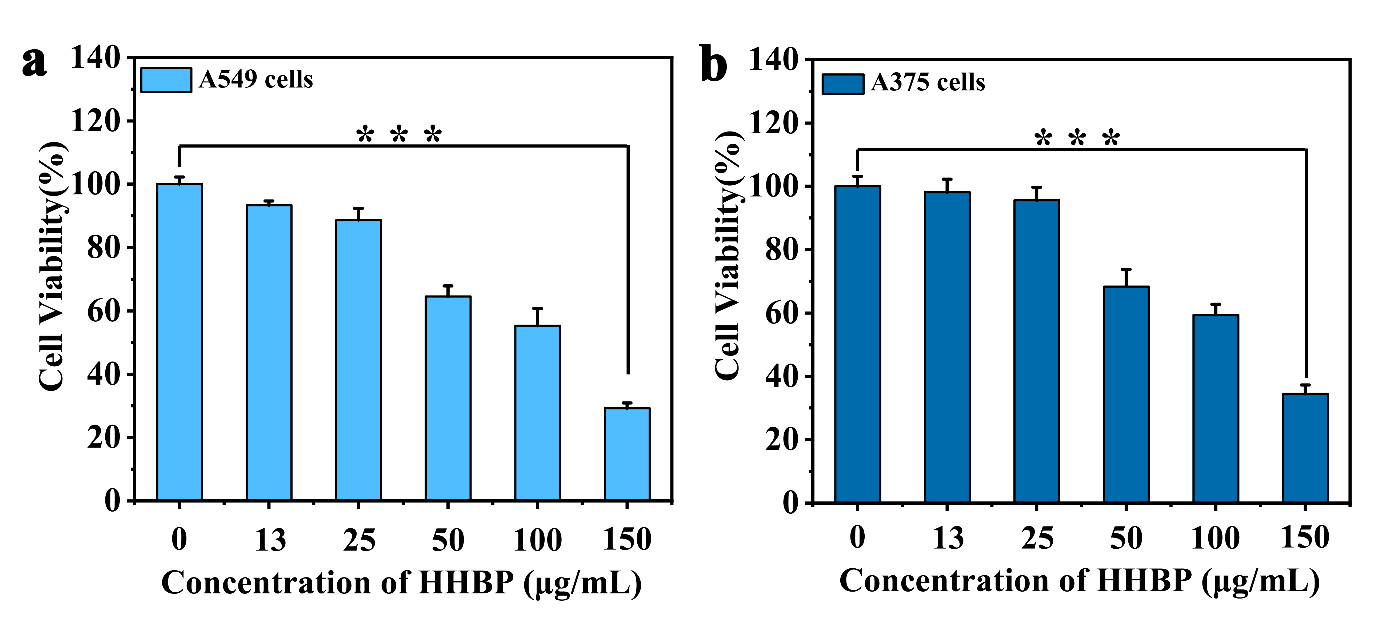


**Fig. S12.** Relative viabilities of **a** A549 and **b** A375 cells after different treatments, quantified by CCK-8 assay (n = 6). ***P < 0.001.


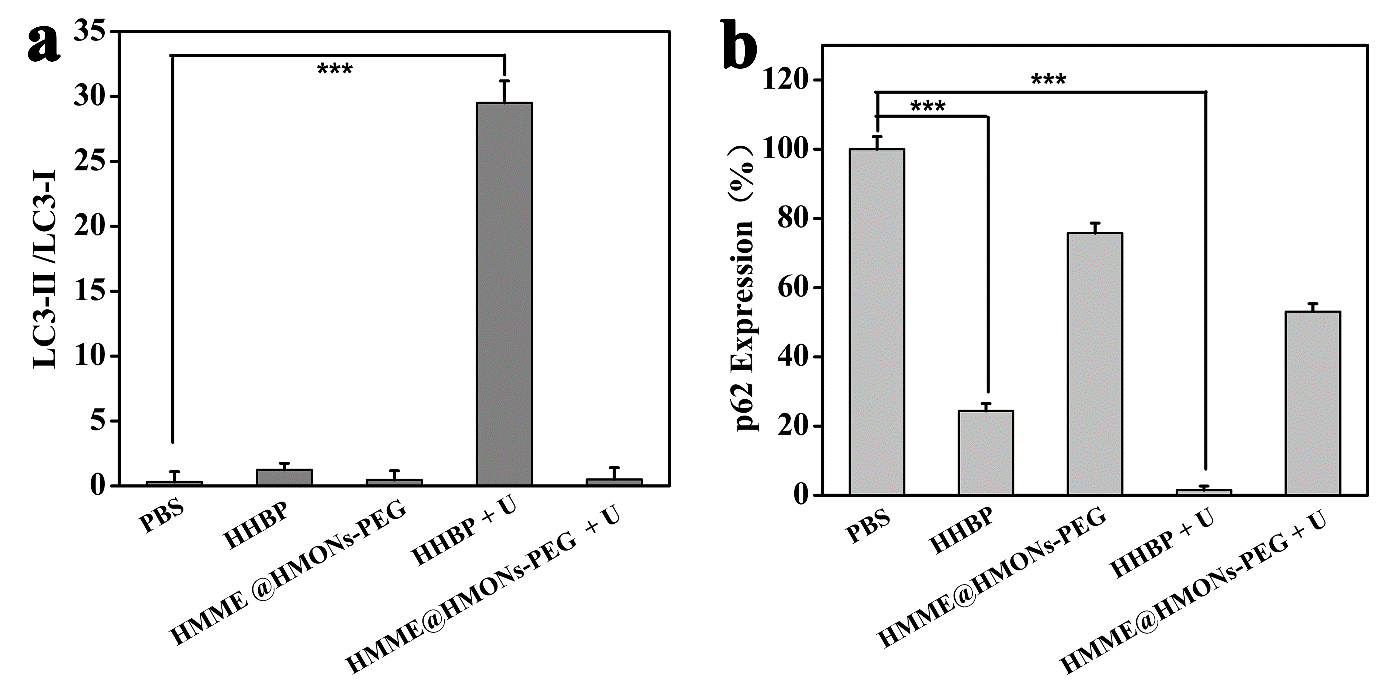


**Fig. S13.** Quantitative analyses of LC3-II/LC3-I **a** and relative p62 **b** expression after different treatments from Western blotting results (see Fig. 3d). ***P < 0.001. Error bars indicate standard deviation (n = 3).


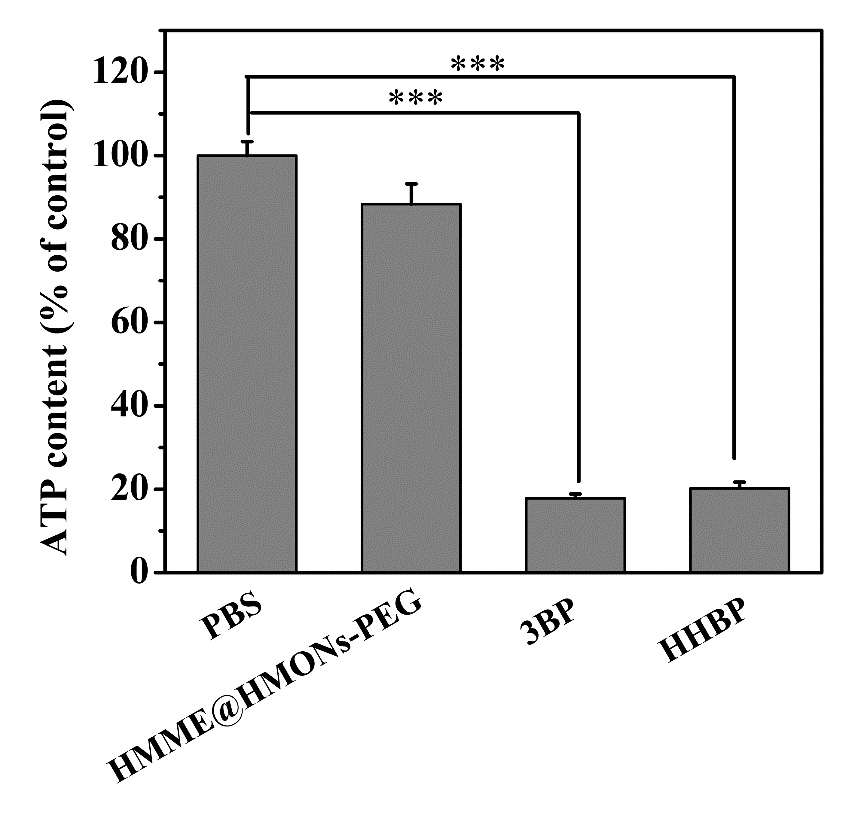


**Fig. S14.** ATP levels in 4T1 cells after different treatments. The data are shown as mean ± SD (n = 3).


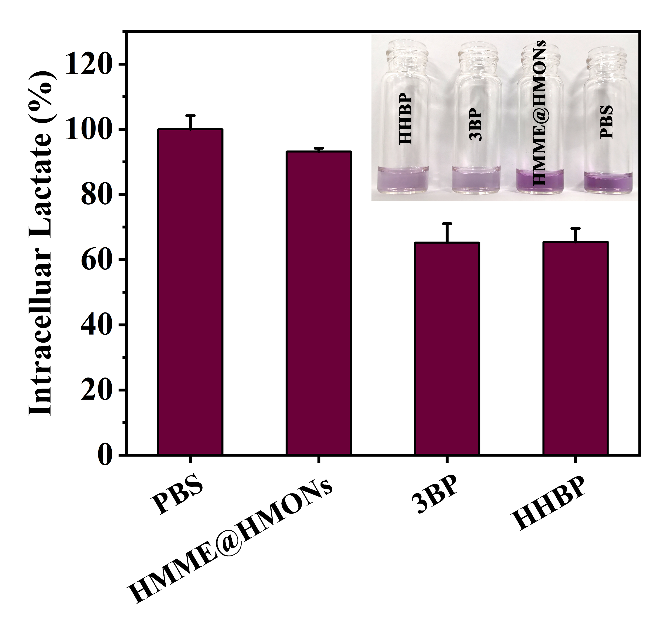


**Fig. S15.** Intracellular lactic acid content of 4T1 cells after incubation with different nanoparticles for 6 h (n = 3).


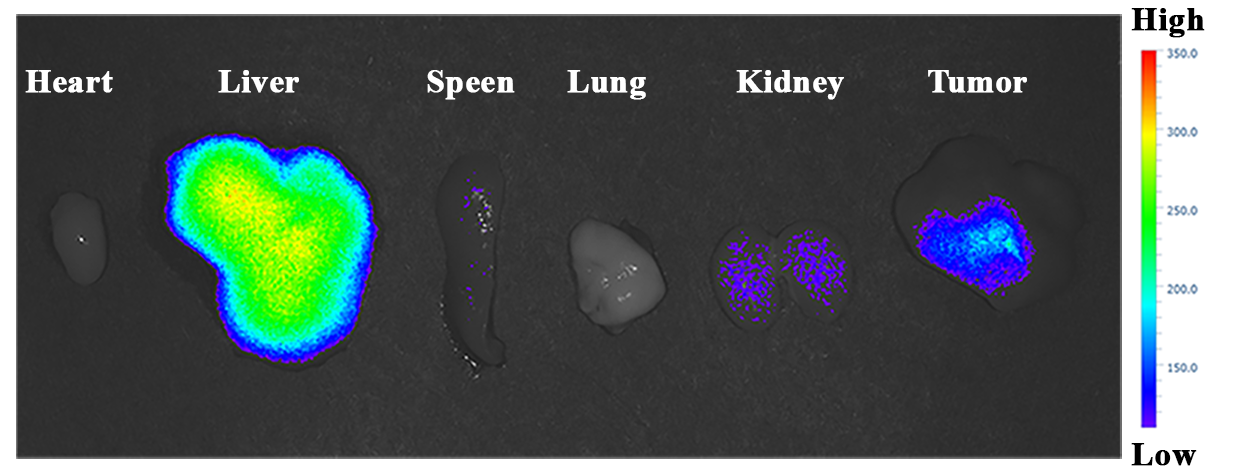


**Fig. S16.** *Ex vivo* fluorescence imaging of the tumor and the major organs including liver, spleen, kidney, heart, and lung, collected from the mice at 24 h post-injection.





**Fig. S17.** Blood circulation lifetime of HHBP after intravenous injection into mice (n = 3).


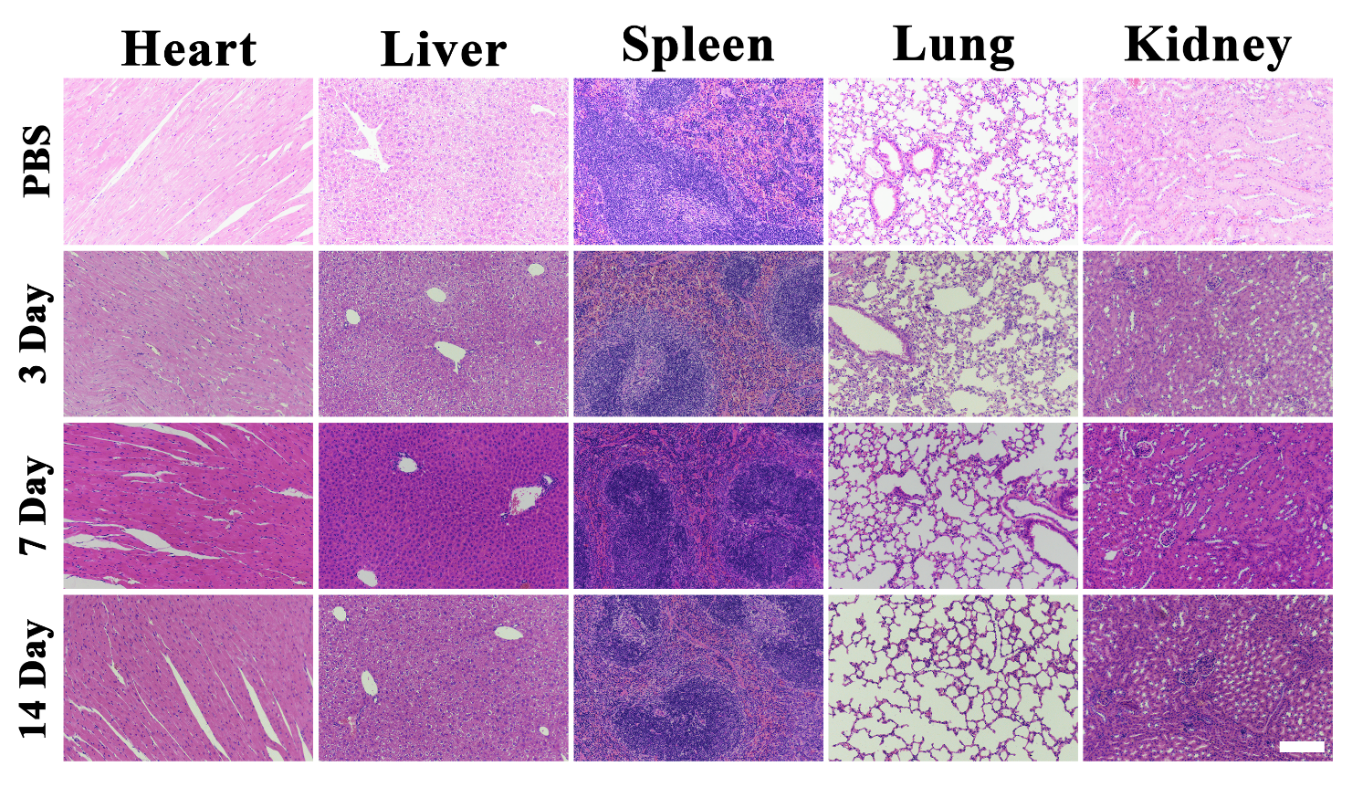


**Fig. S18.** H&E stained histological images of major organs (heart, liver, spleen, lung, and kidney) from mice treated with PBS versus HHBP. Scale bar = 200 μm.


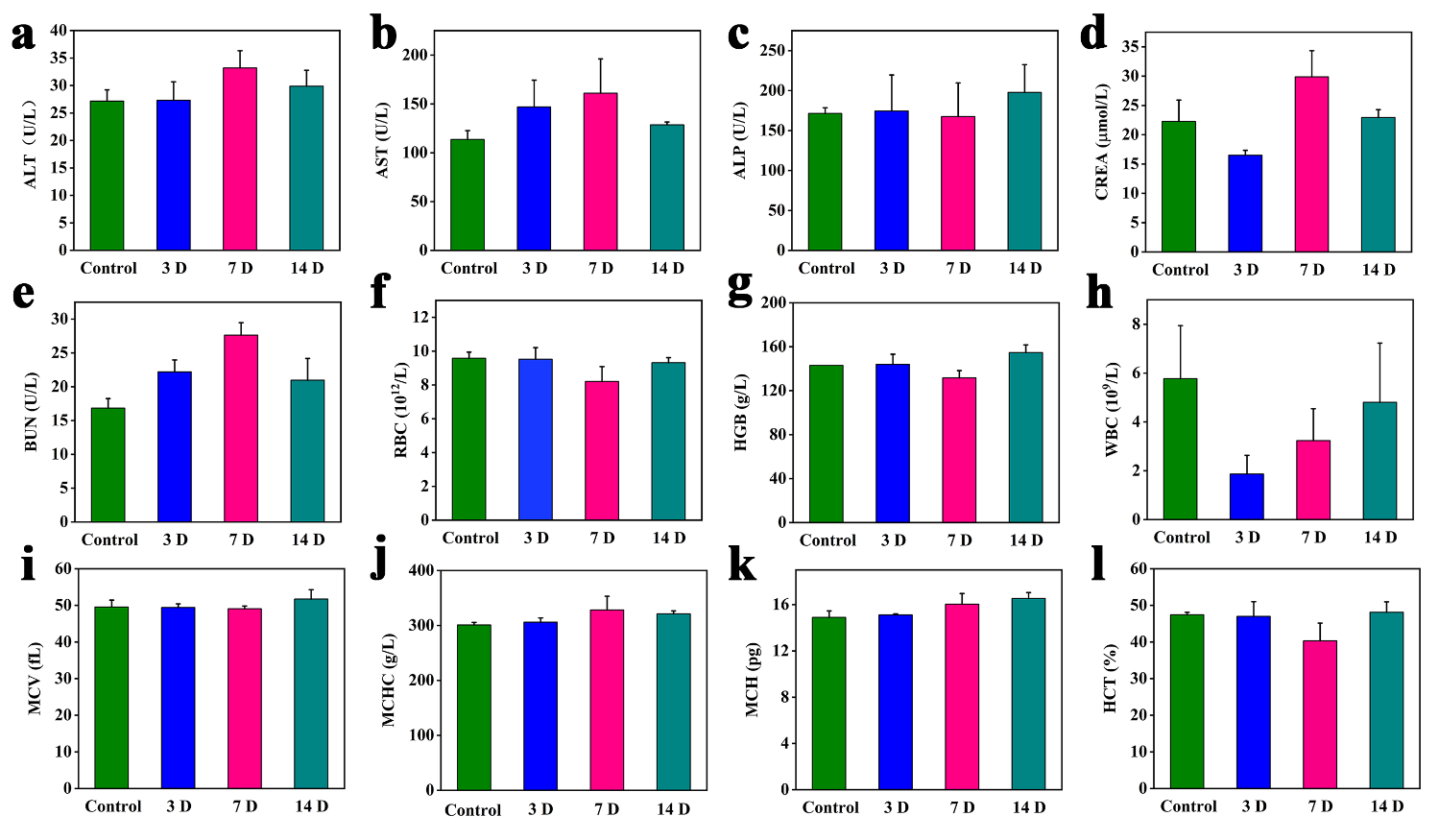


**Fig. S19.** Blood biochemistry and hematology data of Balb/c mice treated with HHBP at different time point after i.v. injection. **a** Alanine aminotransferase (ALT), **b** Aspartate aminotransferase (AST), **c** Alkaline phosphatase (ALP), **d** Creatinine (CREA), **e** Urea nitrogen (BUN), **f** Red blood cells (RBC), **g** Hemoglobin (HGB), **h** White blood cells (WBC), **i** Mean corpuscular volume (MCV), **j** Mean corpuscular hemoglobin concentration (MCHC), **k** Mean corpuscular hemoglobin (MCH), and **l** hematocrit (HCT).
